# Allelic variants of *IRKI* contribute to photosynthetic efficiency by regulating rubisco activase in *Populus*

**DOI:** 10.1093/plphys/kiaf465

**Published:** 2025-09-29

**Authors:** Yongsen Jiang, Dan Wang, Linxia Yang, Yu Cheng, Luying Yang, Tingxuan Zhao, Donghai Zhang, Jiaxuan Zhou, Zitian Li, Yicen Guan, Tailin Ren, Yuling He, Qingzhang Du, Deqiang Zhang, Mingyang Quan

**Affiliations:** State Key Laboratory of Tree Genetics and Breeding, College of Biological Sciences and Technology, Beijing Forestry University, No. 35, Qinghua East Road, Beijing 100083, P. R. China; National Engineering Research Center of Tree Breeding and Ecological Restoration, College of Biological Sciences and Technology, Beijing Forestry University, No. 35, Qinghua East Road, Beijing 100083, P. R. China; Key Laboratory of Genetics and Breeding in Forest Trees and Ornamental Plants, Ministry of Education, College of Biological Sciences and Technology, Beijing Forestry University, No. 35, Qinghua East Road, Beijing 100083, P. R. China; State Key Laboratory of Tree Genetics and Breeding, College of Biological Sciences and Technology, Beijing Forestry University, No. 35, Qinghua East Road, Beijing 100083, P. R. China; National Engineering Research Center of Tree Breeding and Ecological Restoration, College of Biological Sciences and Technology, Beijing Forestry University, No. 35, Qinghua East Road, Beijing 100083, P. R. China; Key Laboratory of Genetics and Breeding in Forest Trees and Ornamental Plants, Ministry of Education, College of Biological Sciences and Technology, Beijing Forestry University, No. 35, Qinghua East Road, Beijing 100083, P. R. China; State Key Laboratory of Tree Genetics and Breeding, College of Biological Sciences and Technology, Beijing Forestry University, No. 35, Qinghua East Road, Beijing 100083, P. R. China; National Engineering Research Center of Tree Breeding and Ecological Restoration, College of Biological Sciences and Technology, Beijing Forestry University, No. 35, Qinghua East Road, Beijing 100083, P. R. China; Key Laboratory of Genetics and Breeding in Forest Trees and Ornamental Plants, Ministry of Education, College of Biological Sciences and Technology, Beijing Forestry University, No. 35, Qinghua East Road, Beijing 100083, P. R. China; State Key Laboratory of Tree Genetics and Breeding, College of Biological Sciences and Technology, Beijing Forestry University, No. 35, Qinghua East Road, Beijing 100083, P. R. China; National Engineering Research Center of Tree Breeding and Ecological Restoration, College of Biological Sciences and Technology, Beijing Forestry University, No. 35, Qinghua East Road, Beijing 100083, P. R. China; Key Laboratory of Genetics and Breeding in Forest Trees and Ornamental Plants, Ministry of Education, College of Biological Sciences and Technology, Beijing Forestry University, No. 35, Qinghua East Road, Beijing 100083, P. R. China; State Key Laboratory of Tree Genetics and Breeding, College of Biological Sciences and Technology, Beijing Forestry University, No. 35, Qinghua East Road, Beijing 100083, P. R. China; National Engineering Research Center of Tree Breeding and Ecological Restoration, College of Biological Sciences and Technology, Beijing Forestry University, No. 35, Qinghua East Road, Beijing 100083, P. R. China; Key Laboratory of Genetics and Breeding in Forest Trees and Ornamental Plants, Ministry of Education, College of Biological Sciences and Technology, Beijing Forestry University, No. 35, Qinghua East Road, Beijing 100083, P. R. China; State Key Laboratory of Tree Genetics and Breeding, College of Biological Sciences and Technology, Beijing Forestry University, No. 35, Qinghua East Road, Beijing 100083, P. R. China; National Engineering Research Center of Tree Breeding and Ecological Restoration, College of Biological Sciences and Technology, Beijing Forestry University, No. 35, Qinghua East Road, Beijing 100083, P. R. China; Key Laboratory of Genetics and Breeding in Forest Trees and Ornamental Plants, Ministry of Education, College of Biological Sciences and Technology, Beijing Forestry University, No. 35, Qinghua East Road, Beijing 100083, P. R. China; State Key Laboratory of Tree Genetics and Breeding, College of Biological Sciences and Technology, Beijing Forestry University, No. 35, Qinghua East Road, Beijing 100083, P. R. China; National Engineering Research Center of Tree Breeding and Ecological Restoration, College of Biological Sciences and Technology, Beijing Forestry University, No. 35, Qinghua East Road, Beijing 100083, P. R. China; Key Laboratory of Genetics and Breeding in Forest Trees and Ornamental Plants, Ministry of Education, College of Biological Sciences and Technology, Beijing Forestry University, No. 35, Qinghua East Road, Beijing 100083, P. R. China; State Key Laboratory of Tree Genetics and Breeding, College of Biological Sciences and Technology, Beijing Forestry University, No. 35, Qinghua East Road, Beijing 100083, P. R. China; National Engineering Research Center of Tree Breeding and Ecological Restoration, College of Biological Sciences and Technology, Beijing Forestry University, No. 35, Qinghua East Road, Beijing 100083, P. R. China; Key Laboratory of Genetics and Breeding in Forest Trees and Ornamental Plants, Ministry of Education, College of Biological Sciences and Technology, Beijing Forestry University, No. 35, Qinghua East Road, Beijing 100083, P. R. China; State Key Laboratory of Tree Genetics and Breeding, College of Biological Sciences and Technology, Beijing Forestry University, No. 35, Qinghua East Road, Beijing 100083, P. R. China; National Engineering Research Center of Tree Breeding and Ecological Restoration, College of Biological Sciences and Technology, Beijing Forestry University, No. 35, Qinghua East Road, Beijing 100083, P. R. China; Key Laboratory of Genetics and Breeding in Forest Trees and Ornamental Plants, Ministry of Education, College of Biological Sciences and Technology, Beijing Forestry University, No. 35, Qinghua East Road, Beijing 100083, P. R. China; State Key Laboratory of Tree Genetics and Breeding, College of Biological Sciences and Technology, Beijing Forestry University, No. 35, Qinghua East Road, Beijing 100083, P. R. China; National Engineering Research Center of Tree Breeding and Ecological Restoration, College of Biological Sciences and Technology, Beijing Forestry University, No. 35, Qinghua East Road, Beijing 100083, P. R. China; Key Laboratory of Genetics and Breeding in Forest Trees and Ornamental Plants, Ministry of Education, College of Biological Sciences and Technology, Beijing Forestry University, No. 35, Qinghua East Road, Beijing 100083, P. R. China; State Key Laboratory of Tree Genetics and Breeding, College of Biological Sciences and Technology, Beijing Forestry University, No. 35, Qinghua East Road, Beijing 100083, P. R. China; National Engineering Research Center of Tree Breeding and Ecological Restoration, College of Biological Sciences and Technology, Beijing Forestry University, No. 35, Qinghua East Road, Beijing 100083, P. R. China; Key Laboratory of Genetics and Breeding in Forest Trees and Ornamental Plants, Ministry of Education, College of Biological Sciences and Technology, Beijing Forestry University, No. 35, Qinghua East Road, Beijing 100083, P. R. China; State Key Laboratory of Tree Genetics and Breeding, College of Biological Sciences and Technology, Beijing Forestry University, No. 35, Qinghua East Road, Beijing 100083, P. R. China; National Engineering Research Center of Tree Breeding and Ecological Restoration, College of Biological Sciences and Technology, Beijing Forestry University, No. 35, Qinghua East Road, Beijing 100083, P. R. China; Key Laboratory of Genetics and Breeding in Forest Trees and Ornamental Plants, Ministry of Education, College of Biological Sciences and Technology, Beijing Forestry University, No. 35, Qinghua East Road, Beijing 100083, P. R. China; State Key Laboratory of Tree Genetics and Breeding, College of Biological Sciences and Technology, Beijing Forestry University, No. 35, Qinghua East Road, Beijing 100083, P. R. China; National Engineering Research Center of Tree Breeding and Ecological Restoration, College of Biological Sciences and Technology, Beijing Forestry University, No. 35, Qinghua East Road, Beijing 100083, P. R. China; Key Laboratory of Genetics and Breeding in Forest Trees and Ornamental Plants, Ministry of Education, College of Biological Sciences and Technology, Beijing Forestry University, No. 35, Qinghua East Road, Beijing 100083, P. R. China; State Key Laboratory of Tree Genetics and Breeding, College of Biological Sciences and Technology, Beijing Forestry University, No. 35, Qinghua East Road, Beijing 100083, P. R. China; National Engineering Research Center of Tree Breeding and Ecological Restoration, College of Biological Sciences and Technology, Beijing Forestry University, No. 35, Qinghua East Road, Beijing 100083, P. R. China; Key Laboratory of Genetics and Breeding in Forest Trees and Ornamental Plants, Ministry of Education, College of Biological Sciences and Technology, Beijing Forestry University, No. 35, Qinghua East Road, Beijing 100083, P. R. China; State Key Laboratory of Tree Genetics and Breeding, College of Biological Sciences and Technology, Beijing Forestry University, No. 35, Qinghua East Road, Beijing 100083, P. R. China; National Engineering Research Center of Tree Breeding and Ecological Restoration, College of Biological Sciences and Technology, Beijing Forestry University, No. 35, Qinghua East Road, Beijing 100083, P. R. China; Key Laboratory of Genetics and Breeding in Forest Trees and Ornamental Plants, Ministry of Education, College of Biological Sciences and Technology, Beijing Forestry University, No. 35, Qinghua East Road, Beijing 100083, P. R. China

## Abstract

Photosynthesis directly determines plant biomass accumulation by controlling carbon flow and energy input. Thus, increasing photosynthetic efficiency is a promising approach for boosting plant growth and yield. However, the genetic basis of photosynthesis in perennial woody plants remains largely unknown, and the causative alleles warrant comprehensive investigation. Here, we performed a genome-wide association study (GWAS) on photosynthetic traits in a natural population of Chinese white poplar (*Populus tomentosa*). We identified *inflorescence and root apices receptor-like kinase-interacting protein* (*IRKI*) as a causative gene of photosynthesis that is significantly associated with the activity of rubisco activase (Rca). The seventh leaves of *PtoIRKI*-overexpression (OE) plants exhibited a 27.77% increase in net photosynthetic rate (Pn), a 31.42% rise in starch content, and a 16.83% expansion in leaf area compared to wild-type plants, whereas *ptoirki*-knockdown (KD) plants displayed the opposite phenotypes. Further analyses indicated that PtoIRKI interacted with PtoRca to enhance Rca activity, leading to increases in the activation state of ribulose bisphosphate carboxylase oxygenase (rubisco) and photosynthetic efficiency. Importantly, we identified an elite haplotype, *PtoIRKI^hap2^*, which exhibited higher *PtoIRKI* expression and Pn than *PtoIRKI^hap1^*. Finally, we found that homeodomain-leucine zipper protein 1 (PtoHB1) specifically bound to the *PtoIRKI^hap2^* promoter, thereby promoting *PtoIRKI* expression and photosynthetic efficiency, as validated by integrating machine learning models and molecular experiments. Our results shed light on the molecular mechanism through which *PtoIRKI* modulates photosynthetic efficiency. We also provide an excellent haplotype module, *PtoHB1-PtoIRKI^hap2^-PtoRca*, that can be used to improve the photosynthesis of woody plants via molecular breeding.

## Teaser

The discovery of a gene–haplotype module that enhances photosynthesis in poplar reveals molecular targets for improving biomass in perennial woody plants.

## Introduction

In nature, solar energy is converted into chemical energy through photosynthesis, which transforms inorganic carbon into biological compounds, producing more than 100 billion tons of dry biomass annually (Jin et al. 2023). The resulting carbon flow and energy input are utilized in various aspects of plant growth and development, including seed germination, nutrient absorption, root expansion, mechanical strength maintenance, and canopy formation (Yeh et al. 2022). However, the efficiency of converting intercepted solar radiation into plant biomass by photosynthesis remains elusive. Thus, enhancing photosynthetic efficiency and capacity has become a major research frontier in modern plant breeding (Long et al. 2015). Perennial woody plants have long growth cycles and complex photosynthetic systems, and investigating how to improve photosynthetic efficiency and enhance the accumulation of photosynthetic products in trees holds great potential for boosting biomass energy production, including wood and lignocellulose (Wang et al. 2018).

Photosynthesis consists of 2 critical stages: light and dark reactions. The former involves the absorption and transfer of light energy, electron transfer, and proton transmembrane processes, along with the production of NADPH and ATP. The latter, also known as carbon fixation, utilizes ATP and NADPH produced by the light reaction to transform CO_2_ into carbohydrates through the Calvin–Benson (CB) cycle (Mullineaux 2014; Nikkanen et al. 2021). Previous studies have shown that the photosynthetic efficiency of leaves is influenced by 3 major processes: ribulose bisphosphate carboxylase oxygenase (rubisco) activity, which is reflected by the maximum carboxylation rate; photosynthetic electron transport, which determines the ability to regenerate the rubisco substrate RuBP; and triose phosphate utilization, which drives carbohydrate synthesis from precursors in the CB cycle (Ma et al. 2024). Numerous genetic engineering studies have reported the significant roles of rate-limiting enzymes and causative photosynthetic genes in improving photosynthetic activity (Chida et al. 2007; Simkin et al. 2017; An et al. 2020). Thus, understanding the genetic regulatory networks governing the core photosynthetic stages and critical photosynthetic components is conducive to the genetic improvement of plant photosynthesis.

Rubisco is a key enzyme involved in CO_2_ fixation. It plays a primary rate-limiting role in photosynthesis, making it a critical target for genetic improvement, facilitated by its slow turnover rate and low substrate affinity (Sharwood 2017). The rubisco complex consists of 8 small subunits (RbcS) and 8 large subunits (RbcL), and its activity is strictly regulated by its chaperone rubisco activase (Rca) enzyme, belonging to the AAA+ protein family. Rca interacts with the N-terminal of the highly conserved RbcL subunits in higher plants, activating its catalytic activity in an ATP-dependent manner, thereby initiating carbon fixation by rubisco (Baginsky 2016; Ng et al. 2020). Previously, heterologous transformation of rice *Rca* or introduction of a highly active *OsRca* promoter into maize significantly improved photosynthetic efficiency by increasing the activation state of rubisco (Zhang et al. 2019; Feng et al. 2023). Several transcription factors (TFs) that regulate *Rca* expression have been identified in crops. For example, Grain number, plant height, and heading date 2 (Ghd2) binds to the CACA motif in the *OsRca* promoter through its CCT domain, thereby upregulating *OsRca* expression, which leads to enhanced rice yield (Fan et al. 2022). However, these studies have only preliminarily reported the potential molecular mechanisms underlying the *Rca* gene, and the complex regulatory networks of *Rca* in photosynthesis remain elusive.

Photosynthesis is a complex biological process precisely regulated by multiple genes, ultimately influencing various quantitative traits in plants (Wang et al. 2020). With the rapid advancement of molecular techniques, genome-wide association study (GWAS) has become a powerful tool to identify causative molecular markers across the genome associated with traits. Previous studies have identified genes responsible for key steps in photosynthesis in model plants. For example, a GWAS identified that the acid phosphatase gene *acid phosphatase gene* (*ACP2*) is associated with photosynthetic phosphorus use efficiency (PPUE) in rice. *OsACP2* regulates serine metabolism to adapt to Pi starvation, and the elite haplotype *OsACP2^v5A^* in its promoter results in higher gene expression, PPUE, tiller numbers, and biomass compared to *OsACP2^v5G^* (Liu et al. 2024). Due to the long-life cycle and excellent environmental adaptability, perennial woody plants have accumulated abundant genetic variation, thereby making GWAS a powerful tool for investigating genetic variation in leaf development, leaf morphology, and photosynthetic pathways in trees (Xiao et al. 2020; Li et al. 2024; Zhou et al. 2024). However, since the existing studies lack sufficient experiment-based regulatory network investigation of genes, the genetic basis of photosynthesis remains poorly understood in perennial woody plants, and the genetic effects of causative alleles require comprehensive exploration.

In this study, we investigated the genetic basis of traits related to photosynthetic efficiency by conducting a GWAS on 300 unrelated individuals from a natural population of Chinese white poplar (*Populus tomentosa*). We identified the most significant signal, located in the promoter of a gene encoding the inflorescence and root apices receptor-like kinase-interacting protein (PtoIRKI), which was associated with Rca activity. We found that PtoIRKI interacts with PtoRca to enhance rubisco activity, thereby promoting photosynthetic efficiency and starch accumulation in chloroplasts. The peak signal and a 12-bp Insertion and Deletion (InDel) in the *PtoIRKI* promoter showed strong linkage disequilibrium (LD), with the photosynthetic efficiency of the accessions being divided into 2 haplotype groups. Interestingly, individuals carrying the *PtoIRKI^hap2^* elite haplotype possessed a binding motif of homeodomain-leucine zipper protein 1 (PtoHB1), which enhanced *PtoIRKI* expression, resulting in higher Rca activity, photosynthetic efficiency, and starch content compared to *PtoIRKI^hap1^*. Our study provides valuable genetic insights for improving photosynthetic efficiency through molecular breeding strategies in trees.

## Results

### Genetic basis of photosynthetic efficiency in *P. tomentosa*

To investigate the genetic architecture underlying photosynthetic efficiency, we assessed 17 photosynthetic traits in 3 trait categories, using an association population of 300 individuals of *P. tomentosa*. The coefficient of variation (CV) of photosynthetic traits ranged from 0.278 (Leaf type ferredoxin NADP^+^ oxidoreductase, LFNR) to 3.639 (Stomatal conductivity, Cond), with 12 traits exhibiting CV values exceeding 0.5, indicating substantial phenotypic variation in the photosynthetic traits (Supplementary Table S1). Pearson correlation coefficient (PCC) analysis detected a total of 14 pairs with significant correlation (PCC = −0.28 to 0.99, *P* < 0.05). Phenotypes within the same category frequently exhibited significant correlations, with the exception of certain enzymes (Supplementary Fig. S1). These results suggest a significant genetic diversity of photosynthetic traits in the association population, which can be used for further genetic analysis.

**Figure 1.**
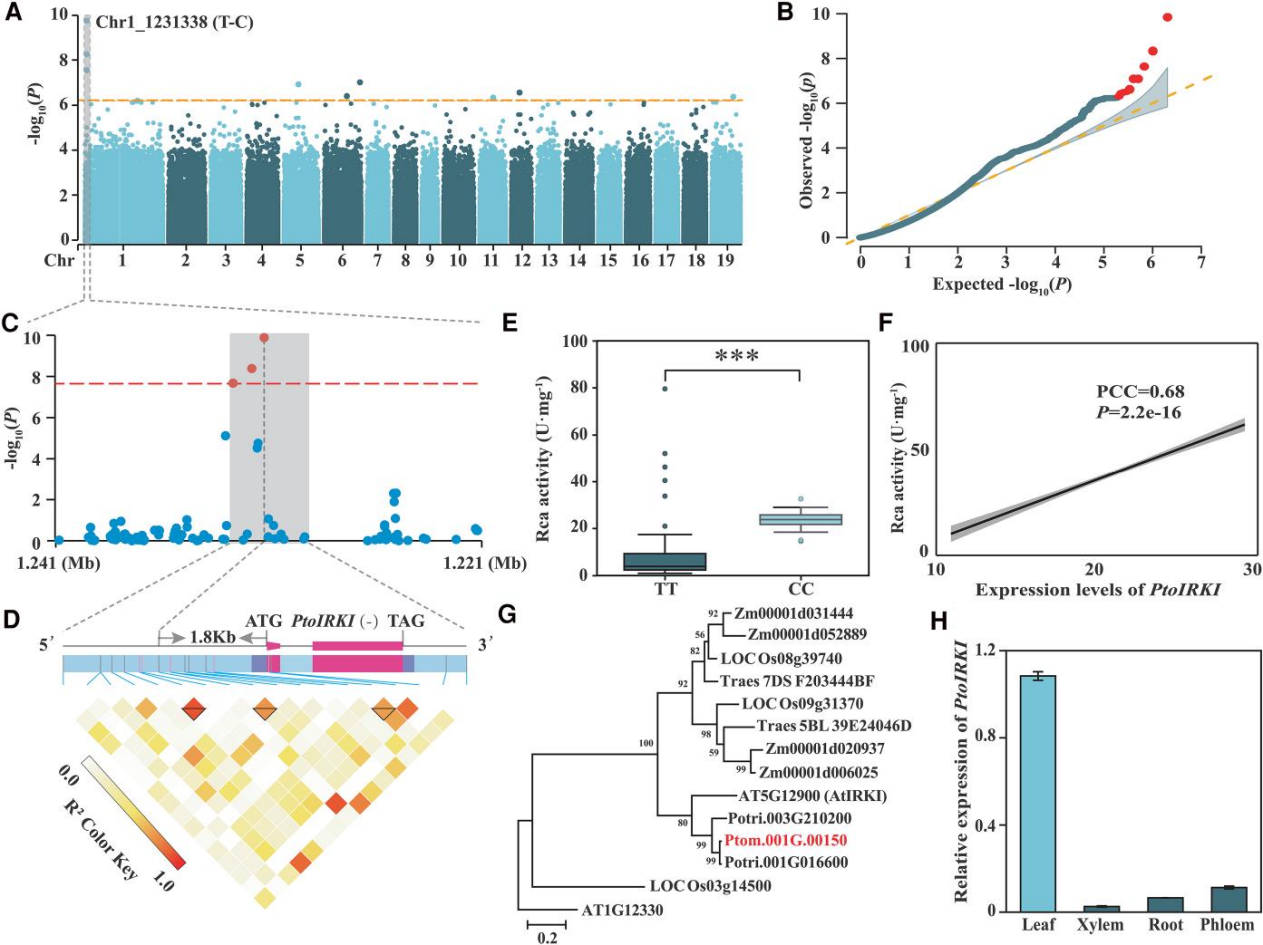
GWAS of the photosynthetic efficiency of a natural population of *P. tomentosa*. **A)** Manhattan plot for GWAS of Rca activity. Virtual horizontal line, Bonferroni-adjusted significance threshold (*P* < 4.94E−07). **B)** QQ plot for GWAS of Rca activity in 300 *P. tomentosa* accessions. Red dots represent SNPs significantly associated with Rca activity. The blue dots represent the observed −log_10_ (*P*), while the dashed line indicates the expected distribution. The shaded area indicates the 95% CI. **C)** Local magnification of GWAS results near the most significant SNP. Red lines and dots represent threshold lines and significant SNPs, respectively. Blue dots represent nonsignificant SNPs. **D)** Gene structure of *PtoIRKI*; the triangle indicates an LD block. **E)** Genotypic effect of Chr1_1231338 on Rca activity in 300 *P. tomentosa* accessions. In box plots, the center line represents the median, the box limits denote the upper and lower quartiles, the whiskers indicate the interquartile range, and the dots are outliers. Significant differences were determined using a Student's *t*-test. ***P < 0.001. **F)** PCC between Rca activity and *PtoIRKI* expression levels in a natural population of *P. tomentosa*. **G)** Phylogenetic tree of PtoIRKI in different species. The phylogenetic tree was performed using the full-length protein sequences of PtoIRKI and its homologs in *T. aestivum*, *O. sativa*, *Z. mays*, *A. thaliana*, *P. trichocarpa*, and *P. tomentosa*. The tree is drawn to scale, with branch lengths measured in the number of substitutions per site. **H)** Expression levels of *PtoIRKI* across different tissues of *P. tomentosa,* as determined via RT-qPCR. The leaves were taken as a reference. Data are means ± standard deviation (SD) (*n* = 3).

Next, we performed a GWAS for photosynthetic traits using 2,024,059 single-nucleotide polymorphisms (SNPs) from the population genome resequencing. In total, 180, 10, and 4 SNPs were significantly associated with enzyme activities of SBP, Rca, and rubisco, respectively, while no SNP was significantly associated with the remaining 14 traits (*P* < 4.94 × 10^−7^; Fig. 1, A and B and Supplementary Table S2). Each SNP explained phenotypic variance (*R*^2^) ranging from 10.54% to 21.95% (average *R*^2^ = 14.24%). These SNPs were annotated to 74 candidate genes (Supplementary Table S2). The most significant SNP, Chr1_1231338 (T/C) (*P* = 1.3 × 10^−10^), located 1.8 kb upstream of *Ptom.001G.00150* coding sequence (CDS), was significantly associated with Rca activity (Fig. 1, C to E). The Rca activity of individuals with the TT allele at SNP Chr1_1231338 was significantly higher than that of individuals with the CC allele in the natural population (*P* = 2.84 × 10^−4^; Fig. 1E). Moreover, the expression levels of *Ptom.001G.00150* were significantly positively correlated with Rca activity (PCC = 0.68, *P* = 2.20 × 10^−6^; Fig. 1F). Phylogenetic analysis indicated that *Ptom.001G.00150* is a homolog of *AtIRKI*, which participates in meristem maintenance and differentiation (Fig. 1G) (Hattan et al. 2004); therefore, we named it *PtoIRKI*. Expression pattern analyses by reverse transcription quantitative polymerase chain reaction (RT-qPCR) showed that *PtoIRKI* expression was highest in leaves compared to vascular tissues (Fig. 1H). Therefore, we speculated that *PtoIRKI* may be the causative gene associated with changes in leaf photosynthesis in the *P. tomentosa* population.

### *PtoIRKI* positively contributes to photosynthetic efficiency

**Figure 2.**
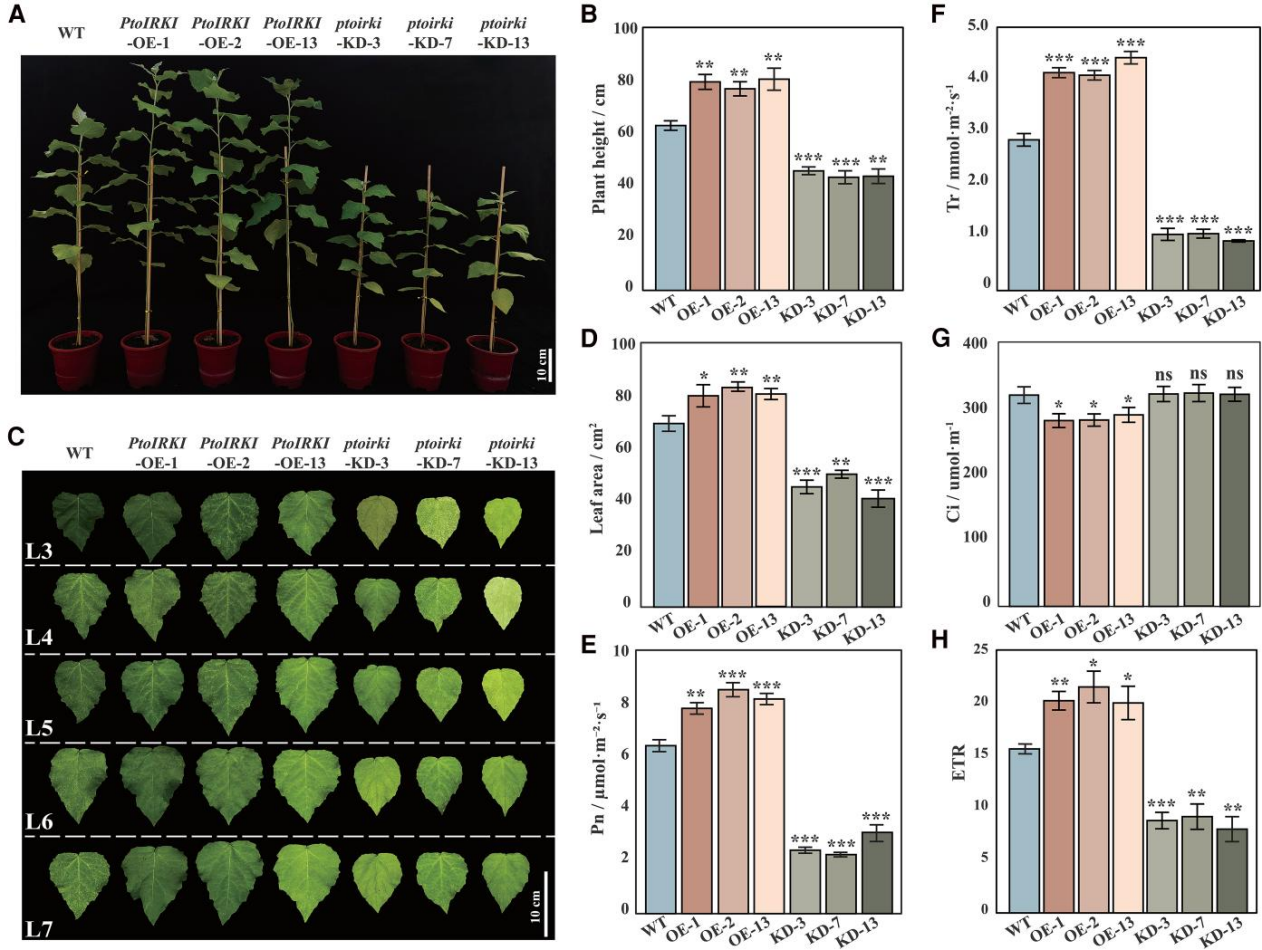
Phenotypes of *PtoIRKI* transgenic lines. **A)** Phenotypic representations of WT, OE, and KD plants. **B)** Plant height of WT, OE, and KD plants (*n* = 3). **C)** Phenotypes of the third to seventh leaves (L3 to L7) of greenhouse-cultivated individuals 3 mo after transfer to soil. Images were digitally extracted for comparison. **D)** The area of the seventh leaves of WT, OE, and KD plants (*n* = 3). **E)** to **H)** Net photosynthesis **(E**, Pn**)**, transpiration rate **(F**, Tr**)**, intercellular CO_2_ concentration **(G**, Ci**)**, and electron transfer rate **(H**, ETR**)** of the seventh leaves of WT, OE, and KD plants (*n* = 3). Error bars are ±SD; significant differences were determined using Student’s *t*-tests. ***P < 0.001; **P < 0.01; *P < 0.05; ns, no significant difference.

To test the biological functions of *PtoIRKI*, we generated 3 transgenic lines overexpressing *PtoIRKI* (*PtoIRKI*-OE-1/2/13) and 3 lines with *PtoIRKI* KD (*ptoirki*-KD-3/7/9). RT-qPCR analysis showed that the average expression level of *PtoIRKI*-OE lines and *ptoirki*-KD lines was 3.02-fold higher and 0.11-fold lower than that of their wild-type (WT) counterparts (*P* < 0.05; Supplementary Fig. S2A). Compared to WT plants, *PtoIRKI*-OE plants were significantly taller (by 22.28% to 28.12%), with the seventh leaves increasing in area by 15.11% to 19.63% (*P* < 0.05; Fig. 2, A to D). Similarly, *PtoIRKI*-OE lines exhibited significantly higher photosynthetic parameters, including Pn (27.77%, *P* = 6.58 × 10^−5^) and Tr (37.46%, *P* = 1.41 × 10^−5^), but decreased Ci (17.92%, *P* = 0.023) compared to WT plants (Fig. 2, E to G). Consistent with these results, the electron transport rate (ETR) of *PtoIRKI-*OE plants was significantly higher (by 31.73% vs. WT, *P* = 3.65 × 10^−6^) (Fig. 2H). By contrast, *ptoirki*-KD lines were 27.52% to 31.39% shorter and exhibited seventh leaves that were 27.61% to 40.90% smaller than those of WT plants (*P* < 0.05; Fig. 2, A to D). Correspondingly, Pn and Tr decreased by 59.61% (*P* = 2.12 × 10^−7^) and 64.09% (*P* = 1.83 × 10^−4^), respectively, whereas Ci remained unchanged in KD lines compared to WT (Fig. 2, E to G). ETR in KD plants declined by 44.63% compared to WT (*P* = 1.48 × 10^−7^; Fig. 2H). Interestingly, there was no significant change in Gs in *PtoIRKI-*OE, *ptoirki*-KD, and WT plants (*P* > 0.05; Supplementary Fig. S2B). These results suggest that the expression of *PtoIRKI* positively improved photosynthetic efficiency in poplar leaves.

**Figure 3.**
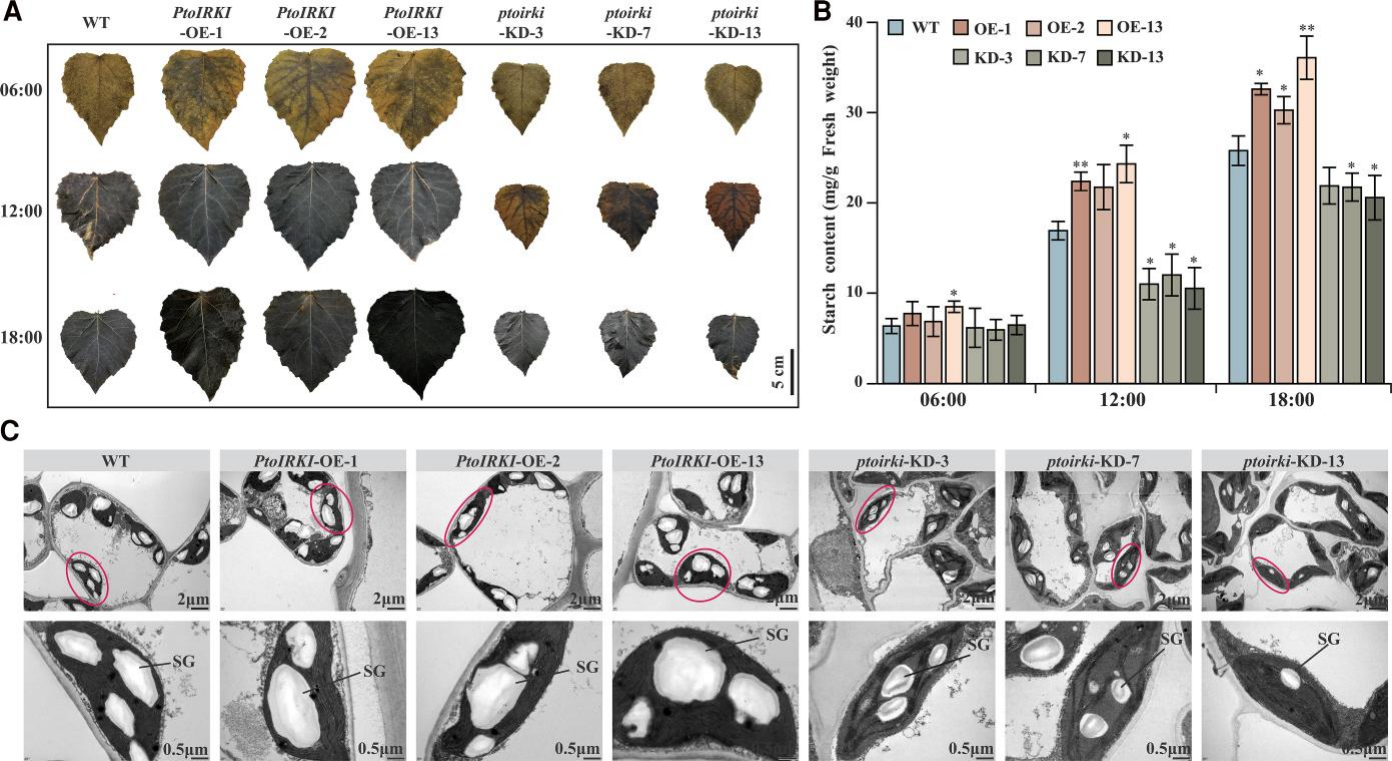
*PtoIRKI* modulates chloroplast ultrastructure and starch levels in poplar. **A)** The starch content at different time points in WT, OE, and KD leaves detected by iodine staining under short-day conditions. The seventh leaves were used. Images were digitally extracted for comparison. **B)** Starch content in the seventh leaves of WT, OE, and KD plants at different time points. Differences were evaluated using 2-tailed Student's *t*-tests. **P < 0.01; *P < 0.05. Data are means ± SD (*n* = 3). **C)** Ultrastructure of chloroplasts in WT, OE, and KD plants. SG, starch granule. The oval highlights the intact chloroplast structure.

Next, we harvested the seventh leaves of plants grown under long-day conditions to examine starch content. A time-course iodine staining analysis demonstrated a progressive increase in starch accumulation from 06:00 to 18:00 across all plants, with *PtoIRKI*-OE plants consistently exhibiting deeper staining intensity than WT and *ptoirki*-KD lines (Fig. 3A). Quantitative analysis revealed that the starch content was slightly higher in *PtoIRKI*-OE lines compared to WT plants at 06:00, and increased by 28.61% to 43.87% in *PtoIRKI*-OE lines compared to WT plants at 12:00 (Fig. 3B). By contrast, the starch content was slightly lower in the KD lines than in WT plants at the beginning of the day; although some starch had accumulated in the KD lines, their starch content was significantly lower than that of the WT plants at 12:00 (*P* = 2.89 × 10^−4^; Fig. 3B). The accumulation of starch reached its peak, with the OE lines being 27.88% (*P* = 1.32 × 10^−3^) higher than WT, while KD plants were 17.02% (*P* = 1.81 × 10^−2^) lower than WT at 18:00 (Fig. 3B).

Because starch is eventually broken down into soluble saccharides for plant growth, the soluble sugar content represents the amount of starch consumed by the plants. Therefore, we determined the soluble sugar content in the transgenic plants at 18:00. Results showed that it did not change significantly in the WT, OE, and KD lines (*P* > 0.05; Supplementary Fig. S2C). Ultrastructural observation showed no discernible differences in chloroplast morphology or spatial distribution among *PtoIRKI*-OE, *ptoirki*-KD, and WT lines; in all cases, chloroplasts formed chain-like arrays positioned adjacent to the cell wall (Fig. 3C). Elliptic starch granules were present in the chloroplasts of all 3 genotypes; granule number did not differ significantly, yet granules were larger in OE chloroplasts and smaller in KD chloroplasts relative to WT (Fig. 3C and Supplementary Fig. S2, D and E), consistent with the results of assays for starch content. In summary, *PtoIRKI* improved photosynthetic efficiency, leading to starch accumulation in the chloroplasts of plants.

### The genetic effects of allelic variants of *PtoIRKI* on photosynthetic efficiency

**Figure 4.**
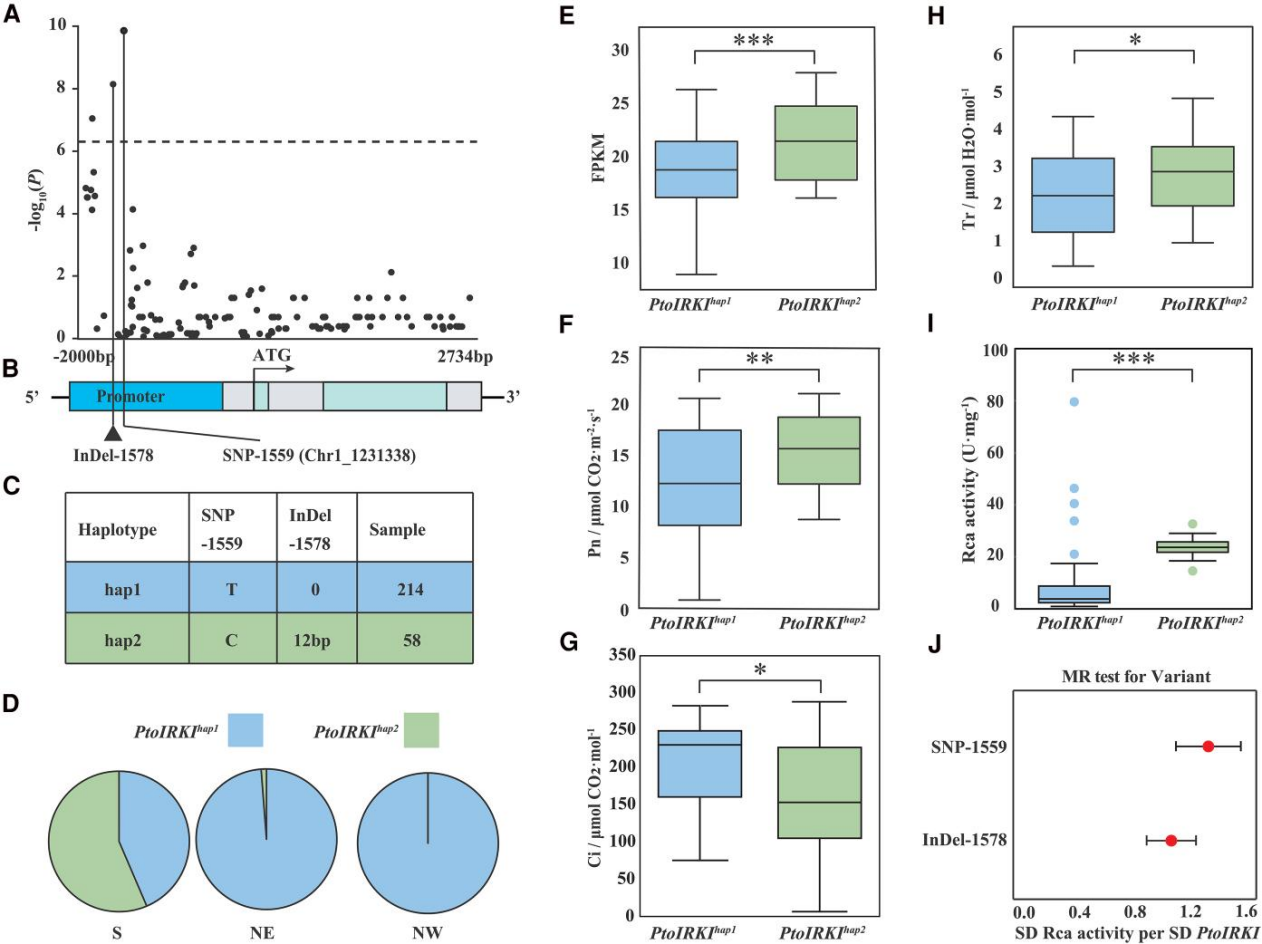
Genetic effects of natural variants in *PtoIRKI.*  **A)** Association analysis between genetic variation in *PtoIRKI* and Rca activity. Each dot represents a genetic marker (SNPs and InDels). Dots and triangles indicate significant SNPs and a 12-bp indel, respectively. **B)** The promoter region and full-length sequence of *PtoIRKI*. **C)**  *PtoIRKI* haplotype groups in natural *P. tomentosa*, categorized by 2 variants. **D)** Distribution of *PtoIRKI* haplotypes in *P. tomentosa* climate regions. NW, NE, and S represent northwest, northeast, and southern climate regions, respectively. **E)** Fragments per kb of transcript per million mapped reads (FPKM) values of *PtoIRKI* between 2 haplotypes in a natural population of *P. tomentosa* (*n* = 272). In box plots, the center line represents the median, the box limits denote the upper and lower quartiles, the whiskers indicate the interquartile range, and the dots are outliers. **F)** to **I)** Box plot of net photosynthetic rate **(F**, Pn**)**, intercellular CO_2_ concentration **(G**, Ci**)**, transpiration rate **(H**, Tr**)**, and distribution of Rca activity **(I)** for each haplotype group. In box plots, the center line represents the median, the box limits denote the upper and lower quartiles, the whiskers indicate the interquartile range, and the dots are outliers. **J)** Estimates of the genetic effects of SNP and InDel in *PtoIRKI* on the expression of *PtoIRKI* and Rca activity. Estimates were derived via MR using inverse variance weighting. Red dots represent estimates, and solid lines represent the range of estimates ± SD. Significant differences were determined using 2-tailed Student’s *t*-tests. ***P < 0.001; **P < 0.01; *P < 0.05.

To determine the molecular basis of the natural variation in *PtoIRKI* of *P*. *tomentosa*, a 4.7-kb genomic DNA fragment encompassing the entire gene body of *PtoIRKI* and its 2-kb promoter region was analyzed in the natural population of 300 *P*. *tomentosa* accessions. In total, 132 SNPs and 24 InDels were identified and subjected to candidate gene-based association analysis (Supplementary Table S3). In the promoter of *PtoIRKI*, a 12-bp InDel (minor allele frequency [MAF] ≥ 0.05) was significantly associated with Rca activity (*P* < 4.94 × 10^−7^; Fig. 4A). Haplotype analysis showed that this InDel (InDel-1578) was in strong LD (*r^2^* > 0.8) with the peak signal (SNP Chr1_1231338) in the *PtoIRKI* promoter identified by GWAS (Fig. 4, A and B). Next, we classified the 272 accessions of *P. tomentosa* into 2 haplotype groups based on 2 common variants (frequency ≥ 5%), *PtoIRKI^hap1^* and *PtoIRKI^hap2^*, excluding 28 individuals from the population due to missing genotypes or heterozygous loci (Fig. 4C). *PtoIRKI^hap1^* was detected mainly in NE and NW accessions, whereas *PtoIRKI^hap2^* occurred mostly in the S accessions, as revealed by allele frequency assessment (Fig. 4D). We also found that the expression levels of *PtoIRKI* in accessions of *PtoIRKI^hap2^* were significantly higher than those in *PtoIRKI^hap1^* (*P* = 2.29 × 10^−6^; Fig. 4E). Subsequently, to evaluate whether the distribution of *PtoIRKI* haplotype frequencies is linked to light-adaptation among distinct subpopulations, we compiled PAR records for the natural range of *P. tomentosa* spanning 2008 to 2018. The dataset revealed that the climatically suitable S region experienced significantly higher PAR values (83.31 W/m^2^, *P* < 0.05), whereas PAR values did not differ significantly between the NE (78.48 W/m^2^) and NW (78.62 W/m^2^) subpopulations (Supplementary Fig. S3A). Moreover, the PAR experienced by individuals carrying *PtoIRKI^hap1^* was 5.48% lower than that of *PtoIRKI^hap2^* carriers (*P* = 2.30 × 10^−7^), suggesting that allelic variation at *PtoIRKI* contributes to regional adaptation to light availability (Supplementary Fig. S3B).

We subsequently evaluated the genetic effects of these polymorphisms of *PtoIRKI* on photosynthetic efficiency, and found that Pn and Tr of *PtoIRKI^hap2^* were significantly higher than those of *PtoIRKI^hap1^*, while Ci was significantly lower than *PtoIRKI^hap1^* (*P* < 0.05); there were no significant differences in Gs between the 2 haplotypes (Fig. 4, F to H; Supplementary Table S4). These results indicate that the photosynthetic efficiency of *PtoIRKI^hap2^* was higher than that of *PtoIRKI^hap1^*. There were no differences in pigment content, but the activity of enzymes involved in carbon fixation (rubisco, Rca, sedoheptulose-1,7-bisphosphatase, and transketolase) was consistently significantly higher in *PtoIRKI^hap2^* (*P* < 0.05; Fig. 4I and Supplementary Table S4). Furthermore, Mendelian randomization (MR) tests showed that the phenotypic variation in Rca was attributable to changes in the expression levels of *PtoIRKI* based on Chr1_12313338 (SNP) and Chr1_1231357 (InDel) (95% CI > 0; Fig. 4J and Supplementary Table S5). Thus, the haplotypes contributed to variation in photosynthetic efficiency, with *PtoIRKI^hap2^* having a greater effect than *PtoIRKI^hap1^*.

### PtoHB1 binds to 12-bp insertion of the *PtoIRKI* promoter and promotes *PtoIRKI* expression

**Figure 5.**
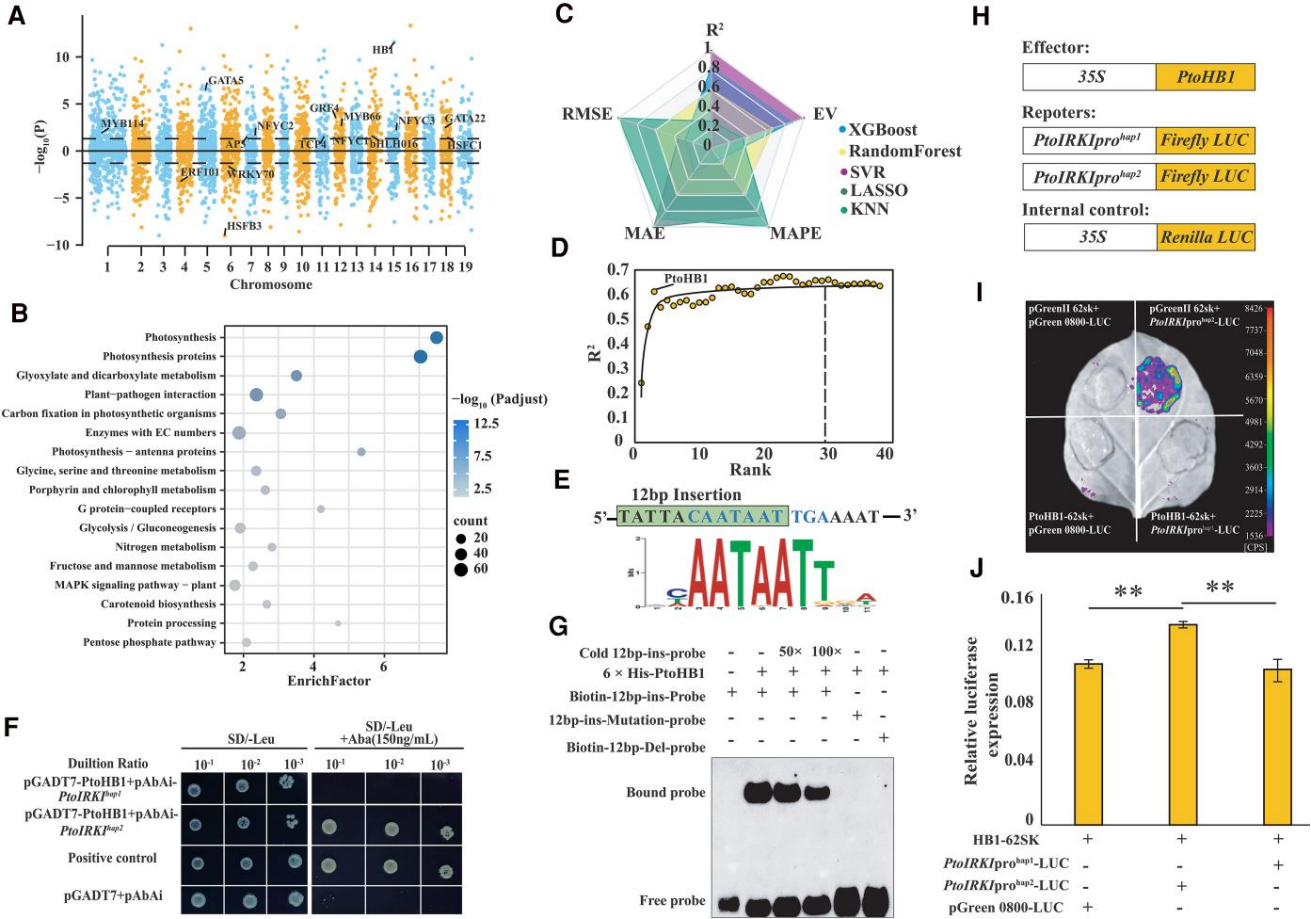
Identification of upstream regulators of *PtoIRKI.*  **A)** Manhattan plot of LMM results for *PtoIRKI* expression. Each point represents a single gene. Genomic positions are plotted on the *X*-axis, and log-transformed false discovery rates of the association between gene expression and *PtoIRKI* expression are plotted on the *Y*-axis. Genes positively or negatively associated with *PtoIRKI* are plotted above or under the bold line, respectively. The dashed line represents the significance level. The genes indicated are TFs with high priority in machine learning. **B)** KEGG analysis of genes highly associated with *PtoIRKI* expression. Sphere size is proportional to gene count, and sphere hue represents the *P-*value. **C)** Performance metrics for XGBoost, random forest, SVR, LASSO, and KNN models of the expression levels of *PtoIRKI*. Each metric is scaled to a range of 0 to 1 to visualize model performance. **D)** Changes in the determination coefficients of model predictive accuracy after sequentially adding the top 40 genes using RFE. Each point represents a gene. The *X*-axis indicates the order in which the genes were added. **E)** Sequence in the green box represents the 12-bp insertion sequence of the *PtoIRKI^hap2^* promoter; light blue sequence represents the most likely binding motif of PtoHB1. The sequence at the bottom is the actual binding motif of *PtoHB1*. **F)** Y1H assay of the binding of PtoHB1 to the *PtoIRKI* promoter. **G)** Binding affinity of PtoHB1 to the promoters of *PtoIRKI^hap1^* and *PtoIRKI^hap2^* was evaluated through an EMSA. + and − indicate the presence and absence of reagents in the lane during protein electrophoresis, respectively. His-labeled probes with the PtoHB1 protein are shown. Bound probe represents the relative binding affinity between PtoHB1 and the *PtoIRKI^hap1^*/*PtoIRKI^hap2^* promoters. **H)** to **J)** Regulation of the activation of *PtoIRKI* by PtoHB1 detected using a dual-luciferase assay (DLRA). The Luciferase/Renilla luciferase ratio was higher in *N. benthamiana* leaves coexpressing *35S*:PtoHB1 and *PtoIRKI^hap2^*-pro-LUC than in those coexpressing *35S*:PtoHB1 and *PtoIRKI^hap1^*-pro-LUC. The scale bar in **I)** represents the fluorescence intensity. The error bars stand for SD of 3 biological replicates, and significant differences were determined using Student's *t*-tests, **P < 0.01.

To investigate the potential gene regulatory networks of *PtoIRKI* involved in photosynthesis, we used a linear mixed model (LMM), which detected 2,338 genes (including 144 TFs) that were linearly correlated with *PtoIRKI* expression (FDR < 0.05; Fig. 5A). Kyoto Encyclopedia of Genes and Genomes (KEGG) pathway enrichment analysis showed that these genes were primarily enriched in photosynthesis and carbon fixation (Fig. 5B). To identify the key TFs influencing *PtoIRKI* expression, 5 machine learning models were used. The support vector regression (SVR) model had the highest coefficient of determination (*R*^2^ = 0.62) and explained variance (EV = 0.62) and lowest root mean square error (RMSE = 2.47), mean absolute error (MAE = 1.79), and mean absolute percentage error (MAPE = 0.13), indicating the superior predictive performance of SVR in modeling gene expression data (Fig. 5C; Table 1). Subsequently, the 144 TFs were further prioritized using recursive feature elimination (RFE) to identify upstream regulators of *PtoIRKI*. The test set's determination coefficient (*R*^2^ ≈ 0.6) stabilized upon adding approximately 30 prioritized genes (Fig. 5D). Consequently, these 30 genes were selected as candidate upstream regulators of *PtoIRKI* (Fig. 5D; Supplementary Table S6).

**Table 1.** Predictive performance and rank of machine learning models for PtoIRKI expression

Model	*R* ^2^	Rank	EV	Rank	MAPE	Rank	MAE	Rank	RMSE	Rank	Comprehensive rank
SVR	0.62	1	0.62	1	0.13	1	1.79	1	2.47	1	1
XGBoost	0.52	2	0.54	2	0.14	2	2.11	2	2.78	2	2
RandomForest	0.39	3	0.41	3	0.17	3	2.41	3	3.14	4	3
LASSO	0.04	5	0.00065	5	0.19	4	2.86	5	2.86	3	4
KNN	0.20	4	0.21	4	0.2	5	2.75	4	3.58	5	4

*R* ^2^, coefficient of determination; EV, explained variance; RMSE, root mean square error; MAE, mean absolute error; MAPE, mean absolute percentage error.

The comprehensive ranking of the model is obtained based on all indicators, with the top ranking being considered the best model.

To identify the transcriptional differences between the 2 haplotypes, TF binding site prediction was performed to determine the upstream regulator (Supplementary Table S7) in the *Populus* genome. In total, 40 consistent binding TFs were predicted (*P* < 1 × 10^−5^) in 2 haplotypes, and *PtoIRKI^hap2^* had 3 more specific HD-zip genes at the 12-bp insertion sequence than those in *PtoIRKI^hap1^* (Supplementary Table S7). Of these, *PtoHB1* (*Ptom.015G.00464*) and *PtoIRKI* showed the highest correlation (LMM_*FDR* = 2.68 × 10^−12^), ranking third in the RFE results. Therefore, *PtoHB1* may be an upstream regulator of *PtoIRKI*. The transcriptional activation experiment confirmed that PtoHB1 has transcriptional activation activity (Supplementary Fig. S4), and HB1 was previously reported to be a transcriptional activator involved in leaf development (Aoyama et al. 1995). Sequence analysis revealed that the haplotype block in the *PtoIRKI^hap2^* promoter contains HD-ZIP binding sites, and has a core motif spanning 7 bp of the 12-bp insertion (InDel-1578, strong LD with the lead SNP Chr1_1231338), which is absent in the *PtoIRKI^hap1^* promoter (Fig. 5E). In addition, we further verified that 5 priority genes have a high PCC with *PtoIRKI* expression (| PCC | = 0.37 to 0.66; Supplementary Table S8). We also examined the interaction between the top 5 priority genes and the 2 haplotype promoters of *PtoIRKI* through the yeast 1-hybrid (Y1H) assays (Fig. 5F and Supplementary Fig. S5, A and B). The results indicated that only PtoHB1 could bind to the 12-bp insertion in the *PtoIRKI^hap2^* promoter, and none of the genes could bind to the *PtoIRKI^hap1^* promoter (Fig. 5F and Supplementary Fig. S5, A and B). Therefore, we hypothesized that PtoHB1 could be responsible for differences in *PtoIRKI* expression between the 2 distinct alleles.

Next, to verify the molecular interactions between PtoHB1 and *PtoIRKI*, we examined the binding ability between the former and the 2 *PtoIRKI* haplotypes. Electrophoretic mobility shift assays (EMSAs) revealed that PtoHB1 strongly and specifically binds to the promoter of *PtoIRKI^hap2^*, rather than that of *PtoIRKI^hap1^* (Fig. 5G). Next, we investigated the effect of PtoHB1 on the promoter activity of the different *PtoIRKI* haplotypes using the dual-luciferase reporter assays (DLRAs) in *N. benthamiana* leaves. Negative controls were PtoHB1 recombined with *PtoIRKI^hap1^* promoter or without the *PtoIRKI* promoter. Strong luciferase luminescence was observed when *PtoIRKI^hap2^* pro-LUC and PtoHB1 were cotransformed into *N. benthamiana* leaves, and the negative controls did not luminesce (Fig. 5, H to J). These results showed that PtoHB1 enhances the expression of *PtoIRKI^hap2^* by binding to the 12-bp insertion in its promoter, thereby positively regulating photosynthesis.

### Interaction between PtoIRKI and PtoRca in chloroplasts enhances rubisco activity

**Figure 6.**
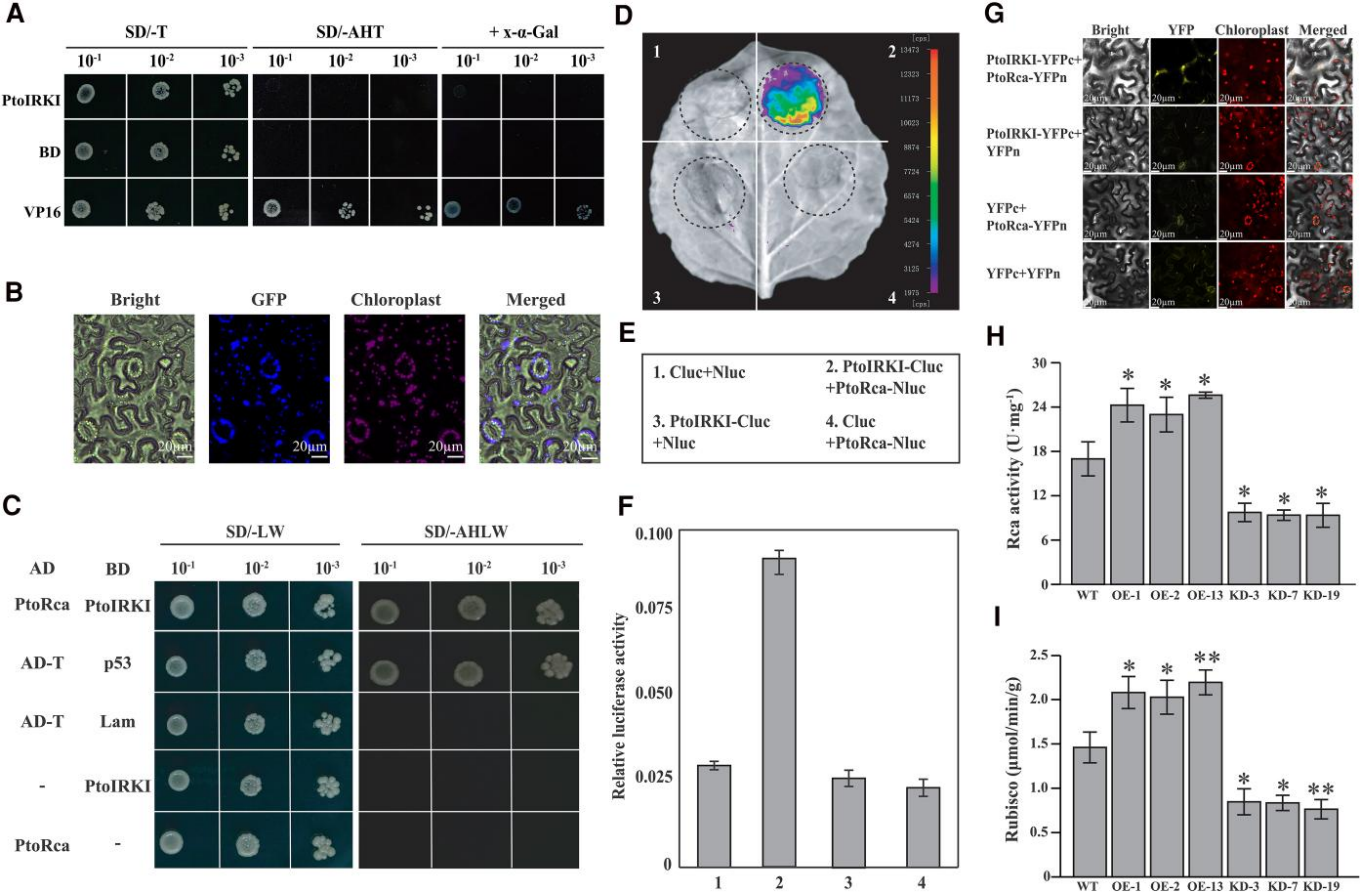
Interaction between PtoIRKI and PtoRca in vitro and in vivo, and determination of enzyme activity in transgenic plants. **A)** Analysis of the transcriptional activation of *PtoIRKI*. **B)** Subcellular localization of PtoIRKI demonstrated by transient expression of *35S*: PtoIRKI-GFP in *N. benthamiana* leaves (*N* = 3). **C)** Interaction between PtoIRKI and PtoRca in *P. tomentosa* was observed in vitro using a Y2H assay. Empty vectors, pGADT7 or pGBKT7, served as negative controls (*N* = 3). **D)** to **F)** Interaction between PtoIRKI and PtoRca in vivo was investigated using a luciferase bioluminescence imaging assay **(D, E)**. Young *N. benthamiana* leaves were subjected to a firefly luciferase complementation assay **(F)**. Error bars indicate SD from 3 biological replicates. **G)** In vivo interaction between PtoIRKI and PtoRca according to bimolecular fluorescence complementation assays. **H)** Rca and **I)** rubisco activity in WT, OE, and KD plants (*n* = 3). Error bars are ± SD. Differences were evaluated using 2-tailed Student’s *t*-tests. *P < 0.05; **P < 0.01.

Next, we investigated the underlying mechanism of PtoIRKI affecting photosynthetic efficiency. Given that PtoIRKI lacks a typical transcriptional regulation domain, we hypothesized that it may interact with photosynthesis-related genes to modulate the activity of photosynthetic proteins. Transcriptional activation experiments showed that PtoIRKI had no transcriptional activation activity (Fig. 6A). To examine the subcellular localization of PtoIRKI, we fused PtoIRKI with green fluorescent protein (GFP) and performed a transient expression assay in *N. benthamiana* leaves. Results indicated that the PtoIRKI-GFP fusion protein was localized to the chloroplast (Fig. 6B).

To further clarify the molecular functions of PtoIRKI, yeast 2-hybrid (Y2H) screening was conducted to identify proteins that interact with PtoIRKI. Several putative candidates were identified, including *Ptom.008G.00582*, an ortholog of *AtRca* (*AT2G39730*; Fig. 6C; Supplementary Table S9). Moreover, a firefly luciferase complementation assay showed strong luciferase signals in *N. benthamiana* leaves (Fig. 6, D to F), while a biomolecular fluorescence complementation (BiFC) assay revealed the YFP signal in the chloroplasts of epidermal cells (Fig. 6G). These results confirmed the interactions between PtoIRKI and PtoRca in chloroplasts. To examine whether these interactions enhanced rubisco activity, we measured the activities of Rca and rubisco enzyme in fully expanded seventh leaves of *PtoIRKI*-OE, *ptoirki*-KD, and WT plants. Compared to WT, rubisco and Rca activities increased by 34.93% and 42.93%, respectively, in OE plants, whereas the same activities decreased by 44.18% and 44.16% in KD lines (Fig. 6, H and I). These results suggested that PtoIRKI enhances rubisco activity through its interaction with Rca, thereby accelerating the carbon fixation process and promoting starch production, which in turn facilitates the growth of poplar.

## Discussion

Photosynthesis, a crucial process for plant growth and development, is controlled by multiple genetic factors at various levels and is highly sensitive to environmental conditions. Identifying allelic variants that affect photosynthetic efficiency and understanding the regulatory mechanisms of causative genes are crucial for the molecular breeding of high-productivity tree varieties (Pan et al. 2018). In this study, we integrated genomic, transcriptomic, and phenotypic data to dissect the genetic basis and regulatory network of photosynthetic efficiency using association mapping and machine learning. We identified *PtoIRKI* as a causative regulator that enhances photosynthetic efficiency by affecting Rca activity. Specifically, we identified a molecular module, *PtoHB1-PtoIRKI^hap2^-PtoRca*, which plays a crucial role in regulating photosynthetic efficiency in perennial woody plants.

### Systems genetics analysis revealed a positive regulator of Rca activity in *P. tomentosa*

Photosynthesis is an extremely complex and sophisticated system, making it challenging to dissect its underlying regulatory mechanisms. Previous studies in model plants have identified a number of genes that regulate photosynthesis, and their functions have been validated through transgenic and molecular biological methods. These genes are involved in increasing chlorophyll content and nitrogen utilization, promoting photosynthetic electron transport, improving chloroplast function, and enhancing related enzyme activities during carbon fixation (Tang et al. 2019 ; Feyissa et al. 2024 ; Zhou et al. 2024). Among these, the interaction between Rca and rubisco directly determines the efficiency of carbon fixation, which is an important target for improving photosynthetic efficiency and biomass. Rca is closely related to rubisco activity, and enhancing its expression and activity can significantly improve the photosynthetic rate of plants under high temperature, strong light, or other adverse conditions, offering a potential target for future responses to climate change (Bi et al. 2017; Qu et al. 2021). *Rca* expression is regulated by trans-acting factors in spinach (Orozco and Ogren 1993), *Arabidopsis* (Liu et al. 1996), potato (Qu et al. 2011), rice (Yang et al. 2012; Fan et al. 2022), and soybeans (Chao et al. 2014; Zhang et al. 2016). Several genes involved in regulating Rca activity have been identified. The phosphorylation of Rca by the plastid-targeted casein kinase 2 at threonine_78_ (Thr_78_) in *Arabidopsis* is associated with Rca inactivation in the dark and a consequent decrease in rubisco carbamylation (Boex-Fontvieille et al. 2014 ; Matiolli et al. 2022). However, genes that regulate the activity of these enzymes in trees are rarely reported.

Based on GWAS, we identified a causative gene involved in the regulation of Rca activity (PtoIRKI) that forms a protein complex with PtoRca. Phylogenetic analysis showed that *PtoIRKI* is homologous with *AtIRKI*, which is involved in meristem maintenance and differentiation (Fig. 1G) (Hattan et al. 2004). Phenotypic analysis showed that *PtoIRKI*-OE plants were taller and had greater leaf areas, whereas *ptoirki*-KD plants were correspondingly shorter and displayed reduced leaf areas (Fig. 2, A to D). Additionally, Pn, ETR, and the activities of Rca and rubisco itself increased in OE plants but decreased in KD lines, indicating that *PtoIRKI* positively contributed to the overall photosynthetic rate of poplar (Fig. 2, E and F and Fig. 6, H and I). Consistent with these trends, OE plants accumulated more starch and possessed larger chloroplastic starch granules, whereas KD plants accumulated less starch and formed smaller granules (Fig. 3). These results indicate that PtoIRKI binds to PtoRca, enhancing its activity, which in turn increases rubisco activity, leading to improved photosynthetic efficiency and ultimately starch accumulation. Thus, *PtoIRKI* is a potential target for future molecular breeding to modulate the carbon cycle and improve photosynthetic efficiency.

### Natural variation in the *PtoIRKI* promoter leads to differences in photosynthetic efficiency in *P. tomentosa*

Reverse genetics studies have identified genes that directly regulate photosynthesis, but the favorable alleles of these genes remain unexplored (Huang and Han 2014). Allelic variants play an important role in environmental adaptation in woody plants by having a profound effect on traits at the genomic level (Fang et al. 2023). In this study, we discovered an LD block encapsulating the SNP Chr1_1231338 and one 12-bp InDel within the *PtoIRKI* promoter (Fig. 4, A to C). This block divided the 278 natural *P. tomentosa* individuals into 2 distinct haplotype groups, and those with *PtoIRKI^hap2^* had higher *PtoIRKI* expression and greater Rca activity (an increase of approximately 135.67%) compared to the *PtoIRKI^hap1^* counterparts (Fig. 4; Supplementary Table S5). The photosynthetic parameters Pn (24.48%), Tr (26.06%), and Ci (30.05%) of *PtoIRKI^hap2^* were significantly greater than those of *PtoIRKI^hap1^*, indicating significant differences in photosynthetic efficiency between the 2 haplotypes (*P* < 0.05; Fig. 4, F to H). Additionally, the 2 haplotypes exhibited contrasting geographic distributions. *PtoIRKI^hap2^* predominated in the S subgroup, whereas *PtoIRKI^hap1^* was enriched in the NE and NW subgroups (Fig. 4D). The S subgroup experiences significantly higher mean PAR than the NE and NW subgroups (*P* < 0.05; Supplementary Fig. S3A), and PAR for *PtoIRKI^hap2^* habitats is 5.79% greater than that of *PtoIRKI^hap1^* (Supplementary Fig. S3B). Taken together, the elevated expression and superior photosynthetic performance of *PtoIRKI^hap2^*, coupled with its prevalence in high PAR regions, suggest that cis-regulatory variants in the *PtoIRKI* promoter facilitate light-adaptation by enhancing rubisco activation under intense light.

Several photosynthetic alleles have been identified in crops, as have elite haplotypes. For example, dominant haplotypes related to chlorophyll content and stay-green traits have been identified in rice, which can be utilized to breed rice varieties with enhanced photosynthetic efficiency (Zhao et al. 2019). However, the mechanisms through which allelic variants affect photosynthetic efficiency have rarely been studied, particularly in perennial woody plants, thereby limiting the application of haplotypes to improve forest productivity. Here, we investigated the underlying mechanisms for differentiation in photosynthetic efficiency between the 2 haplotypes. The haplotype block in the *PtoIRKI^hap2^* promoter contains PtoHB1 binding sites, and its core motif spans 7 bp of the 12-bp insertion, which is absent in the *PtoIRKI^hap1^* promoter (Fig. 5E). Furthermore, Y1H assay, EMSA, and DLRA confirmed that PtoHB1 specifically binds to *PtoIRKI^hap2^*. These results suggest that PtoHB1 binds to the *PtoIRKI^hap2^* promoter to promote its expression, thereby improving photosynthetic efficiency (Fig. 5, F to J).

### The *PtoHB1-PtoIRKI^hap2^-PtoRca* module positively regulates photosynthetic efficiency in *P. tomentosa*

Revealing the regulatory relationships among genes will help to clarify the complex regulatory mechanisms of photosynthetic pathways, which can lead to the genetic improvement of photosynthetic efficiency via genetic engineering. Coexpression network analysis is a conventional method for identifying gene regulatory networks (Langfelder and Horvath 2008). However, it relies on correlation analysis of gene expression and is challenged by noise interference and high-dimensional data when dealing with complex biological systems. As a result, the constructed networks may not accurately reflect the actual gene regulatory mechanisms. Machine learning approaches offer an effective alternative, by automatically identifying potential inter-gene interactions and regulatory mechanisms from large-scale gene expression databases in a data-driven manner, particularly in complex gene regulatory networks (Xiao et al. 2016). For example, in a previous study, a regulatory network of expression quantitative trait loci (eQTL) hot genes associated with cotton seed oil levels was constructed by integrating various machine learning models, and the positive regulation of TFs *NAC13* and *SCL31* in relation to seed oil levels was experimentally validated (Tan et al. 2022). In the present study, we identified PtoHB1 as an upstream regulator of *PtoIRKI* by integrating 5 machine learning models to screen and predict promoter binding motifs. The interaction between PtoHB1 and the *PtoIRKI* promoter was validated through molecular biological experiments. These results support the possibility of identifying key genes using machine learning approaches.

**Figure 7.**
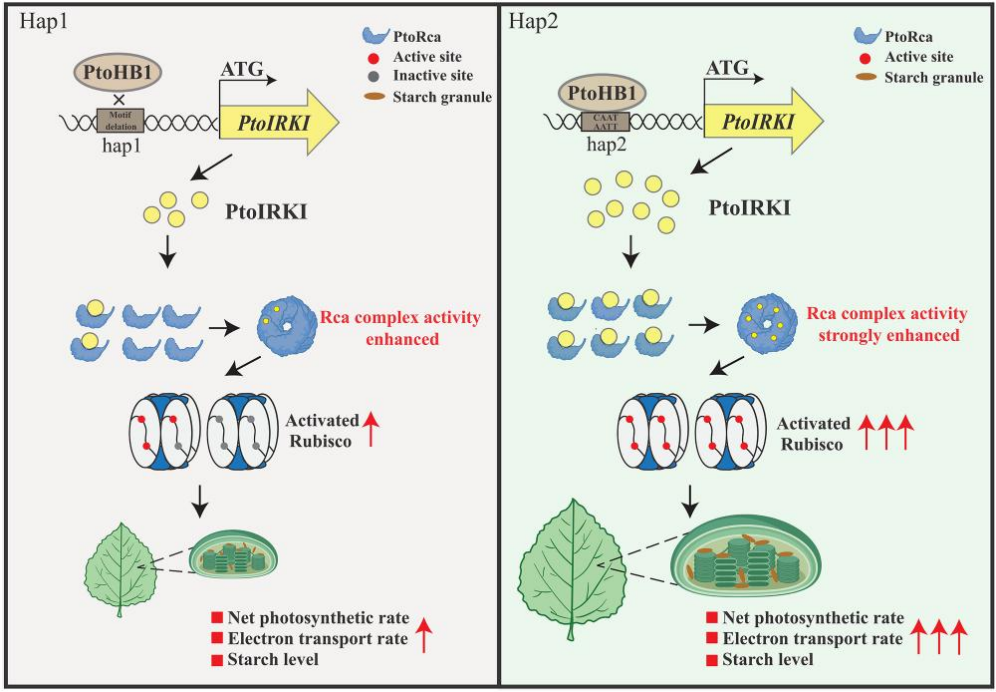
Molecular mechanism underlying the effect of *PtoRca* on photosynthetic efficiency and an elite haplotype module contributing to photosynthetic efficiency in *P. tomentosa.* Allelic variants in the promoter of *PtoIRKI* affect gene expression. PtoHB1 specifically binds to the *PtoIRKI^hap2^* promoter to enhance *PtoIRKI* expression. PtoIRKI interacts with PtoRca to promote rubisco activity, which enhances photosynthetic efficiency and increases the accumulation of starch (Right). In contrast, PtoHB1 does not bind to the *PtoIRKI^hap1^* promoter, resulting in lower *PtoIRKI* expression and weaker Rca activity, thereby reducing the activation state of rubisco (Left). Therefore, *PtoHB1-PtoIRKI^hap2^-PtoRca* module is an elite haplotype module contributing to photosynthetic efficiency in *Populus.*

Rca has different isoforms across different species (Caruana et al. 2022), each with distinct enzymatic activity and transcriptional levels (Perdomo et al. 2021), implying that genomic variation significantly influences Rca. For example, eQTL and reporter analysis identified 2- and 14-bp insertions in the Zm-Rcaβ promoter, which increased its expression (Zhang et al. 2019). Similarly, SNPs in the Rca promoter affect gene expression in soybeans (Chao et al. 2014). In light of our findings, we propose a cascaded allele-specific model of *PtoHB1-PtoIRKI^hap2^-PtoRca* for photosynthetic efficiency, which regulates rubisco activity and starch accumulation (Fig. 7). Although PtoIRKI interacts with PtoRca and enhances Rca ability to activate rubisco, the validation of specific interaction domains will be critical for understanding how PtoIRKI enhances Rca activity, which would be considered in future study. In addition, natural variants in the *PtoIRKI* promoter region affect its ability to bind TFs, and PtoHB1 specifically binds to the *PtoIRKI^hap2^* promoter, resulting in higher photosynthetic efficiency. Overall, this module could present an optimal molecular tool for the genetic enhancement of photosynthetic efficiency and biomass in forest trees.

## Materials and methods

### Association population and phenotypic data

An association population of 300 unrelated individuals was asexually propagated via root segments in 2011 in Guan Xian County, Shandong Province, China (36°23′N, 115°47′E) in a randomized complete block design with 3 replicates. The sampling population was randomly selected from a collection of 1,047 natural Chinese white poplar (*P. tomentosa*) individuals (Du et al. 2012), representing almost the entire natural distribution of *P. tomentosa* (30 to 40°N, 105 to 125°E). The geographic distribution of these 300 accessions was divided into the southern (S, *n* = 84), northwest (NW, *n* = 108), and northeast (NE, *n* = 108) geographic regions (Fang et al. 2023). We selected 17 photosynthetic traits for GWAS analysis, and the sampling procedures were previously reported (Xiao et al. 2020). These traits were categorized into 3 groups: photosynthetic characteristics, including transpiration rate (Tr), Pn, leaf internal CO_2_ concentration (Ci), and stomatal conductance (Gs); pigment contents, including total chlorophyll (Chl), chlorophyll a (Chla), chlorophyll b (Chlb), and carotene (Car); and the activity of enzymes, including rubisco, Rca, sedoheptulose-1,7-bisphosphatase (SBP), transketolase (TKT), fructose-1,6-bisphosphate aldolase (FDA), ferredoxin (FDX), leaf-type ferredoxin NADP^+^ oxidoreductase (LFNR), ribulose-5-phosphate kinase (Ru5PK), and chloroplast-type fructose-1,6-bisphosphatase (ChlFBP). To avoid environmental heterogeneity in measurement, especially the impact of circadian rhythms on photosynthesis, the photosynthetic characteristics of functional leaves (i.e. the fourth to sixth leaves from the top of the stem) were measured at 9:00 to 11:00 Am on sunny days during the growing season. Subsequently, the leaves were immediately frozen in liquid nitrogen for the subsequent determination of pigment content and enzyme activity. All measurements were performed using 3 biological and technical replications per genotype.

### SNP calling of resequencing data from the association population and population genetics analyses

The methods and clean data used to resequence the association population of 300 accessions were previously described (Xiao et al. 2020). To obtain high-confidence SNPs, the dataset was further filtered using the following parameters: MAF > 0.05, missing rate < 0.2, and heterozygosity < 0.9, resulting in a total of 7,892,737 SNPs for further analysis. Population structure analysis was conducted using ADMIXTURE v.1.3.0 (Alexander et al. 2009). First, SNPs with LD > 0.2 were removed to ensure independence. Next, the population numbers contained in the sample *K* = 1 to 9 were set and input into ADMIXTURE v.1.3.0. The cross-validation error corresponding to each population number was calculated, and the minimum cross-validation error was defined as the best *K* value. *K* = 3 was ultimately selected as the optimal population structure, in line with the clustering results obtained by Du et al. (2012) using simple sequence repeats (SSRs). Independent SNPs (LD > 0.2) were input into the EMMAX software (Kang et al. 2010) to calculate kinship.

### GWAS analysis

Seventeen photosynthetic traits were standardized based on *Z*-scores before association analysis. Outliers were defined as having a |*Z*| ≥ 3, and these values were excluded and coded as not applicable (NA). To ensure the reliability of the association results, SNPs with LD > 0.7 were removed, remaining 2,024,059 SNPs for further analysis. GWAS analysis was conducted using the compressed mixed linear model (cMLM) in TASSEL v5.0 (Yu et al. 2006), with population structure and kinship as covariates. The significance threshold for GWAS was set at *P*  *<* 4.94 × 10^−7^ (1/*n*, where *n* is the number of SNPs) based on Bonferroni-adjusted correction for multiple testing. Genes overlapping within 2 kb upstream or downstream of the GWAS signal were considered candidate genes.

### Phylogenetic tree analysis

To annotate the functions of PtoIRKI, BLASTP searches were performed against the Phytozome database. Homologous genes of *PtoIRKI* from *Triticum aestivum*, *Oryza sativa*, *Zea mays*, *Arabidopsis thaliana*, and *Populus trichocarpa* were used to construct a phylogenetic tree using MEGA 6.0 software with the maximum-likelihood method. We employed 1,000 bootstrap test replicates and the Poisson model (Tamura et al. 2013).

### RT-qPCR analysis

To analyze the expression patterns of *PtoIRKI* in different tissues, total RNA from roots, xylem, phloem, and leaves of *P. tomentosa* clone “1316” plants was extracted. Next, the total RNA was quantified utilizing a NanoDrop spectrophotometer (Thermo Fisher Scientific, Waltham, MA, USA), and its integrity was verified through agarose gel electrophoresis. Reverse transcription was performed using HiScript III RT SuperMix for PCR (+gDNA wiper; Vazyme Biotech, Nanjing, China). Primers were designed using the RealTime qPCR Assay Entry tool (https://sg.idtdna.com/Scitools/Applications/RealTimePCR/default.aspx). All reactions were performed with 3 technical and biological repeats, using the poplar *Actin* gene (accession no. *EF145577*) as the internal control. RT-qPCR was performed using a 7500 Fast Real-time PCR system and ChamQ SYBR qPCR Master Mix (Vazyme Biotech), following the PCR protocol reported by Quan et al. (2021). Relative gene expression compared to the reference (*Actin*) was calculated using the 2^−ΔΔCT^ method, as described previously. The gene-specific primers used for RT-qPCR are listed in Supplementary Table S10.

### Gene cloning and plant transformation

The full-length CDS of *PtoIRKI* without a stop codon and the homologous sequence of about 200 bp were amplified from a *P. tomentosa* “1316” clone, and then cloned into the pCXSN vector, respectively, under the control of the 35S promoter. The resulting plasmids were transformed into *Agrobacterium tumefaciens* strain GV3101 cells, which were then used to transform 84 K poplar (*Populus alba* × *Populus glandulosa*) via the *Agrobacterium*-mediated method (Liu et al. 2014). The relative expression levels of positive plants were detected via RT-qPCR, as previously described. Vectors, clone sequences, and corresponding restriction sites for generating OE and KD of *PtoIRKI* poplar have been listed in Supplementary Table S10.

### Growth conditions and phenotype measurement

Transgenic plants were established on woody plant rooting medium (pH = 5.80 to 6.0) containing 0.3 gL^−1^ timentin, 0.05% indole-butyric acid, 1-naphthaleneacetic acid, 20 gL^−1^ sucrose, 2.17 gL^−1^ 1/2 Murashige and Skoog medium (Phytotech Labs, Lenexa, KS, USA), and 0.7% (w/v) agar. After 30 d, the plants were relocated to a growth chamber at a temperature range of 20 to 25 °C and a 16:8 h light: dark photoperiod. The seventh leaves of 3-mo-old transgenic plants were selected for further phenotypic analysis.

Phenotypic measurements of transgenic lines were taken between 9:00 and 11:00 on sunny days during the growing season to minimize the influence of circadian rhythms on photosynthesis. Leaf area was measured using the IMAGEJ software (National Institutes of Health, Bethesda, MD, USA). Photosynthetic characteristics, including Pn, Cond, Ci, and Tr, were measured using the LI-6800 portable photosynthesis system (Li-COR, Lincoln, NE, USA). The ETR was assessed using the PAM-2500 system (Heinz Walz, Effeltrich, Germany). Soluble sugar levels in the leaves were quantified using commercial kits (Jiancheng Bioengineering Institute, Nanjing, China), following the manufacturer's instructions. Rubisco and Rca enzyme activity levels were measured using enzyme-linked immunosorbent assays, following the manufacturer's instructions (Komin, Suzhou, China). Data compilation was based on at least 3 transgenic lines exhibiting a stable, consistent phenotype. Statistical significance was assessed using 2-tailed Student's *t*-tests, with differences considered significant at *P* < 0.05.

### Starch content assay and transmission electron microscope (TEM) analysis

For iodine staining, the seventh leaves of 3-mo-old plants grown under long-day conditions (16:8 h light:dark) were harvested. Considering that starch accumulates during the day and degrades at night, we conducted qualitative analyses of the starch content in leaves at 06:00, 12:00, and 18:00. The harvested leaves were fixed with FAA (formalin: acetic acid: ethanol, 1: 1: 18) and cleared using a chloral hydrate solution (200 g chloral hydrate, 20 g glycerol, and 50 mL ddH_2_O), following the protocol of Horiguchi et al. (2005). The leaf samples were stained with Lugol solution (Solarbio Science & Technology, Beijing, China) for 5 min, destained in deionized water for 1 to 2 h, and immediately photographed. Each line underwent 3 independent biological measurements, and statistical significance was assessed using 2-tailed Student's *t*-tests, with differences considered significant at *P* < 0.05.

The seventh leaves were also collected for TEM analysis. After cleaning their surfaces with distilled water, tissues approximately 2 mm^2^ in size were excised with a single-sided blade, avoiding the veins. TEM was performed using a JEM-1400 Flash HC instrument (JEOL, Tokyo, Japan) according to the protocol described by Salah et al. (2015) . The number and area of starch granules within each chloroplast were quantified using the IMAGEJ software (Schindelin et al. 2015).

### Sequence variation association and haplotype analysis

To explore the allelic variation of *PtoIRKI* associated with photosynthetic efficiency, a 4.7-kb genomic DNA fragment encompassing the entire gene body of *PtoIRKI* and its 2-kb upstream promoter region was analyzed in the resequenced natural population of 300 *P. tomentosa* accessions. All InDels and SNPs were submitted to TASSEL v5.0 software (Yu et al. 2006) for candidate gene-based association analysis as described above. LD analysis was conducted using LDBlockShow software (Dong et al. 2021) for haplotype detection. Phenotypic differences between accessions carrying 2 haplotypes were tested using 2-tailed Student's *t*-tests (*P* < 0.05). Photosynthetically active radiation (PAR) represents the fraction of solar irradiance available for photosynthesis. The dataset of PAR across the natural distribution range of *P. tomentosa* is provided by the National Tibetan Plateau/Third Pole Environment Data Center (http://data.tpdc.ac.cn), which provides long-term climatic records relevant to photosynthetic adaptation (Tang et al. 2022).

### RNA-sequencing analysis

Leaf tissues for RNA-sequencing analysis were collected from a natural population of 300 *P. tomentosa* accessions. The fragments per kb of transcript per million mapped reads (FPKM) values were used to quantify gene expression levels. Genes meeting the following criteria were considered highly expressed and variable in leaves: expression in >20% of the 300 accessions; average FPKM > 1; 95% quantile more than twice the 5% quantile. In total, 12,801 expressed genes were obtained.

### MR analysis

MR analysis was performed to assess the causal relationships between *PtoIRKI* expression levels and Rca activity. Genetic markers, including SNP and InDel, which showed significant associations with Rca activity, were incorporated. The *MendelianRandomization* package in R (R Core Team, Vienna, Austria) was applied, utilizing the inverse-variance weighting technique to consolidate the impacts of multiple genetic markers, as described by Broadbent et al. (2020). The association was considered positive when the 95% confidence interval (CI) of the estimate ranged from 0 to 1, and negative when it ranged from −1 to 0. If the 95% CI included 0, the association was regarded as nonsignificant (*P* > 0.05).

### Machine learning models construction

Association analysis was performed using the LMM in the CPGEN R package, with the kinship matrix derived from SNPs as a covariate, to examine the relationship between the FPKM (log_2_-transformed) of 12,800 genes (excluding *PtoIRKI*) and *PtoIRKI* expression. Genes with a false discovery rate (FDR) < 0.05 were considered to be associated with *PtoIRKI* expression. Subsequently, based on 144 of these TFs, models including eXtreme Gradient Boosting (XGBoost), random forest, SVR, least absolute shrinkage and selection operator (LASSO), and K-nearest neighbor (KNN) were constructed. The dataset was divided into a training set (70%) and a test set (30%), with *R*^2^, EV, RMSE, MAE, and MAPE of the test set used as performance evaluation metrics. Models with higher *R*^2^ and EV, but lower RMSE, MAE, and MAPE, were considered optimized models. All models were hyperparameter optimized by grid search. After selecting the optimal model, the importance of each gene was evaluated using RFE to rank the genes. All models were trained using the Python machine learning library scikit-learn.

### Y1H assays

Approximately 200-bp fragments of the *PtoIRKI^hap1^* promoter (including the 12-bp deletion) and the *PtoIRKI^hap2^* promoter (including the 12-bp insertion) were cloned into the pBait-AbAi vector using the *Sac*I and *Xho*I restriction sites to investigate the interactions between the top 5 high-priority genes (including PtoHSFB3, PtoGATA5, PtoHB1, PtoTIFY9, and PtoGRF4) in RFE and the 2 promoters. *PtoIRKIpro^hap1^*-pAbAi and *PtoIRKIpro^hap2^*-pAbAi were transferred into yeast cells and coated on SD-Ura plates to verify self-activation activity. AbA concentrations were set at 0, 50, 150, 300, 400, 600, and 800 ng/mL to obtain the optimal concentration for inhibiting self-activation. Then, a recombinant pGADT7-PtoHSFB3/PtoGATA5/PtoHB1/PtoTIFY9/PtoGRF4 construct was generated by amplifying and inserting the CDS into the pGADT7 vector. The Y1H assay was performed using the Matchmaker Gold Y1H Library Screening System (Clontech Laboratories, Mountain View, CA, USA) following the manufacturer's instructions. Each experiment was performed in triplicate. All primers are listed in Supplementary Table S10.

#### EMSA

The EMSA was performed as previously described (Fang et al. 2023). The full-length CDS of *PtoHB1* was inserted into the pET32a vector (Zoonbio, Nanjing, China) to produce recombinant *PtoHB1* in *Escherichia coli* BL21. PtoHB1 fusion proteins were purified using PureCube Ni-NTA Agarose (Cube Biotech, Wayne, PA, USA). DNA motifs representing the *PtoIRKI* promoters were synthesized using an EMSA Probe Biotin Labeling Kit (Beyotime Biotechnology, Shanghai, China), by annealing the forward and reverse complementary oligos containing the 12-bp insertion and deletion. Unlabeled probes were used as competitors. A total of 100 ng protein was added to each binding reaction. The probe sequences were as follows: the 12bp-ins-Probe sequence “TATTACAATAATTGA” contained the 12-bp insertion and the PtoHB1 binding motif; the 12bp-del-Probe sequence “TGA” represented the 12-bp deletion and lacked the PtoHB1 binding motif; and the 12bp-Mutation-Probe sequence “ATTAGCCGCCGTGA” showed that the core 7-bp PtoHB1 binding motif was completely mutated. A reciprocal competitive EMSA was performed to evaluate the binding of recombinant PtoHB1 protein to the *PtoIRKI^hap1^*/*PtoIRKI^hap2^* promoters, using the indicated biotin-labeled probes and unlabeled competitors. For each probe, 50× and 100× excess competitor was added. The vector for EMSAs and the corresponding primers are listed in Supplementary Table S10.

#### DLRA

The effector was generated by inserting the full-length CDS of *PtoHB1* into the *Bam*HI and *Sal*I restriction sites of the pGreen II-62-SK vector. To generate the reporter construct, the promoter fragments of *PtoIRKI^hap1^* or *PtoIRKI^hap2^* were cloned into the pGreen II-0800-LUC vector. *A. tumefaciens* GV3101 cells harboring both the effector and reporter recombinant plasmids were coinjected into the abaxial side of the *Nicotiana benthamiana* leaves. After 48 h in a growth chamber, 3 leaf discs (1 cm in diameter) were harvested, sprayed with 1 mM d-luciferin potassium salt (BN11009; Biorigin, Beijing, China), and incubated in the dark for 5 min. The activity levels of firefly and Renilla luciferase were measured using a dual-luciferase assay kit (Beyotime Biotechnology), following the manufacturer's instructions. The LUC/REN ratios for both treatments and controls were calculated to assess the binding activity of PtoHB1 to the *PtoIRKI^hap1^* and *PtoIRKI^hap2^* promoters. Each experiment was conducted in triplicate. Statistical significance was assessed using a 2-tailed Student’s *t*-test, with differences considered significant at *P* < 0.05. All primers are listed in Supplementary Table S10.

### Transcription activation assays of PtoIRKI and PtoHB1

For the transcription activation assays, the full-length CDS of *PtoIRKI* and *PtoHB1* were amplified using gene-specific primers. The amplified products were inserted into pGBKT7 (Clontech Laboratories), and the resulting recombinant plasmids were introduced into the Y2H strain, respectively. The Matchmaker GAL4-based Two-Hybrid System 3 (Clontech Laboratories) was used for the assays. Each liquid yeast culture was diluted to an optical density at 600 nm (OD_600_) of 0.5, and 3 *µ*L of each dilution was inoculated onto synthetic dextrose medium lacking tryptophan (SD-Trp) for the selection of positive clones. The clones were placed on synthetic dextrose medium lacking Trp, histidine, and adenine (SD-Trp-His-Ade) for the transactivation assays. X-α-gal was used to detect the transcription activation activity of *PtoIRKI* and *PtoHB1*. Three biological replicates were carried out for each experiment (Wang et al. 2024). All primers are listed in Supplementary Table S10.

### Subcellular localization of PtoIRKI

The full-length CDS of *PtoIRKI* without the stop codon was cloned into the pBI121-GFP vector in frame with GFP, generating a PtoIRKI-GFP fusion construct driven by the *35S* promoter (*35S: PtoIRKI*-GFP). Two days after infiltration, GFP fluorescence was detected using a Leica TCS SP8 confocal laser scanning microscope (Leica Biosystems, Nussloch, Germany) with the following properties: laser, 488 nm; intensity, 3%; collection bandwidth, 485 to 560 nm; and gain, 700 V. Chloroplasts were detected with the following properties: laser, 488 nm; intensity, 3%; collection bandwidth, 680 to 700 nm; and gain, 710 V. Chloroplast autofluorescence was used as a control. Each experiment was performed with 3 biological replicates.

### Y2H assays

A prey library was constructed using cDNA pooled from the “1316” clone. The pGBKT7-PtoIRKI bait and prey library plasmids were cotransformed into yeast cells and cultured on synthetic dextrose/-Trp-Leu-His-Ade quadruple dropout medium. Positive yeast clones were isolated and used to amplify the prey inserts. The inserts were further sequenced to identify the genes present in the inserts (Benagen, Wuhan, China). The cDNA of *PtoRca* was cloned into the pGADT7 vector as prey. The pGBKT7-53 and pGADT7-T vectors were used as positive controls, whereas the pGADT7-PtoRca and pGBKT7, pGADT7 and pGBKT7-PtoIRKI, as well as empty vectors, were used as negative controls. Various combinations of activation domain (AD) and binding domain (BD) vectors were cotransformed into Y2H. After growth on synthetic dextrose/-Leu-Trp medium for 4 to 6 d at 30 °C, the clones were transferred to the selective medium (synthetic dextrose/-Leu-Trp-His-Ade) at 30 °C for 3 to 4 d.

### Luciferase complementation imaging assay

A firefly luciferase complementation imaging assay was performed by infiltrating *N. benthamiana* leaves, as previously described (Zhou et al. 2015). The full CDS of *PtoRca* was inserted into the binary vector pCambia1300-NLuc to generate PtoRca-NLuc, while that of *PtoIRKI* was inserted into pCambia1300-CLuc to generate CLuc-PtoIRKI. *Agrobacterium* cells harboring individual constructs were suspended in the infiltration medium to a final concentration with OD_600_ = 0.4 and mixed at a 1:1 ratio prior to leaf infiltration (PtoRca-NLuc:CLuc-PtoIRKI, PtoRca-NLuc:CLuc, NLuc:CLuc-PtoIRKI, and NLuc:CLuc). The plants were kept in the dark for 12 h and then transferred to a growth chamber under normal conditions for an additional 24 h. The infiltrated *N. benthamiana* leaves were sprayed with 100 mm luciferin, incubated in the dark for 10 min, and observed under a low-light, cooled, charge-coupled device imaging apparatus (Lumazone_1300B; Roper Bioscience, Sarasota, FL, USA). Each experiment was performed in triplicate. Statistical significance was assessed using a 2-tailed Student's *t*-test, with differences considered significant at *P* < 0.05. The sequence used to construct the vectors was shown in Supplementary Table S10.

### BiFC assay

PtoRca-NLuc/CLuc-PtoIRKI pairs were selected and mixed to infiltrate *N. benthamiana* leaves. Empty YFPc/empty YFPn, PtoIRKI-YFPc/empty YFPn, and PtoRca-YFPn/empty YFPc were used as negative controls. After 48 h, the yellow fluorescence protein (YFP) signal was observed under a Leica SP8 confocal laser scanning microscope (Leica Biosystems). YFP fluorescence was detected using the following properties: laser, 513 nm; intensity, 3%; collection bandwidth, 520 to 570 nm; and gain, 723 V. Chloroplasts were detected with the following properties: laser, 488 nm; intensity, 4%; collection bandwidth, 680 to 700 nm; and gain, 650 V. A YFP signal was detected in leaves coexpressing PtoIRKI-YFPc and PtoRca-YFPn plasmids. Chloroplast autofluorescence was used as a control. Each experiment was performed in triplicate.

### Statistical analyses

For statistical analyses of *PtoIRKI* haplotypes and transgenic poplar phenotypes, Student's *t*-tests were performed. Differences in PAR among the 3 *P. tomentosa* subpopulations (S, NE, and NW) were analyzed using 1-way ANOVA and Tukey's test. Significant differences were indicated by *P < 0.05, **P < 0.01, or ***P < 0.001, ns, with no significant difference.

## Accession numbers

The CDS data generated in this study are available in the GenBank databases under the accession numbers PX401423, PX401424, and PX401425 for *PtoIRKI*, *PtoHB1*, and *PtoRca*, respectively. The raw data of genome resequencing of 300 *P. tomentosa* individuals have been deposited in the Genome Sequence Archive (GSA) in the BIG Data Center at Beijing Institute of Genomics (BIG), Chinese Academy of Sciences, under the accession number CRA000903, which is publicly accessible at http://bigd.big.ac.cn/gsa/.

## Supplementary Material

kiaf465_Supplementary_Data

## Data Availability

The data underlying this article will be shared on reasonable request to the corresponding author.

## Supplementary data

The following materials are available in the online version of this article.

**Supplementary Figure S1.** Pearson correlation among 17 photosynthetic traits.

**Supplementary Figure S2.** The phenotypes of *PtoIRKI*-OE and *ptoirki*-KD lines.

**Supplementary Figure S3.** PAR across different geographical regions and among *PtoIRKI* haplotypes in *P. tomentosa.*

**Supplementary Figure S4.** Analysis of the transcriptional activation of *PtoHB1.*

**Supplementary Figure S5.** Y1H assays of the top 4 (excluding PtoHB1) high-priority genes in the machine learning regression model with 2 haplotype promoters of *PtoIRKI*.

**Supplementary Table S1.** Phenotypic variation of 17 photosynthetic traits in the association population of *P. tomentosa*.

**Supplementary Table S2.** Details of significant SNPs associated with photosynthetic traits in the association population of *P. tomentosa*.

**Supplementary Table S3.** Variants in the genomic region of *PtoIRKI*.

**Supplementary Table S4.** Differences in photosynthetic traits among different *PtoIRKI* haplotypes.

**Supplementary Table S5.** MR test for variants.

**Supplementary Table S6.** The 30 prioritized genes identified using LMM and RFE.

**Supplementary Table S7.** Predicting upstream regulators based on the promoters of *PtoIRKI^hap1^* and *PtoIRKI^hap2^*.

**Supplementary Table S8.** PCCs between the top 5 prioritized genes and *PtoIRKI* expression.

**Supplementary Table S9.** Putative proteins interacting with PtoIRKI based on Y2H assay screening library.

**Supplementary Table S10.** The oligonucleotide sequences of primers used in this study.
