# Supplementary material for: Allelic variants of IRKI contribute to photosynthetic efficiency by regulating rubisco activase in Populus
Source: Plant Physiol. 2025 Sep 29;199(2):kiaf465. doi: 10.1093/plphys/kiaf465 (PMC12541374; doi:10.1093/plphys/kiaf465)
Supplement: kiaf465_Supplementary_Data [file kiaf465_supplementary_data.zip › Supplementary Data.pdf]

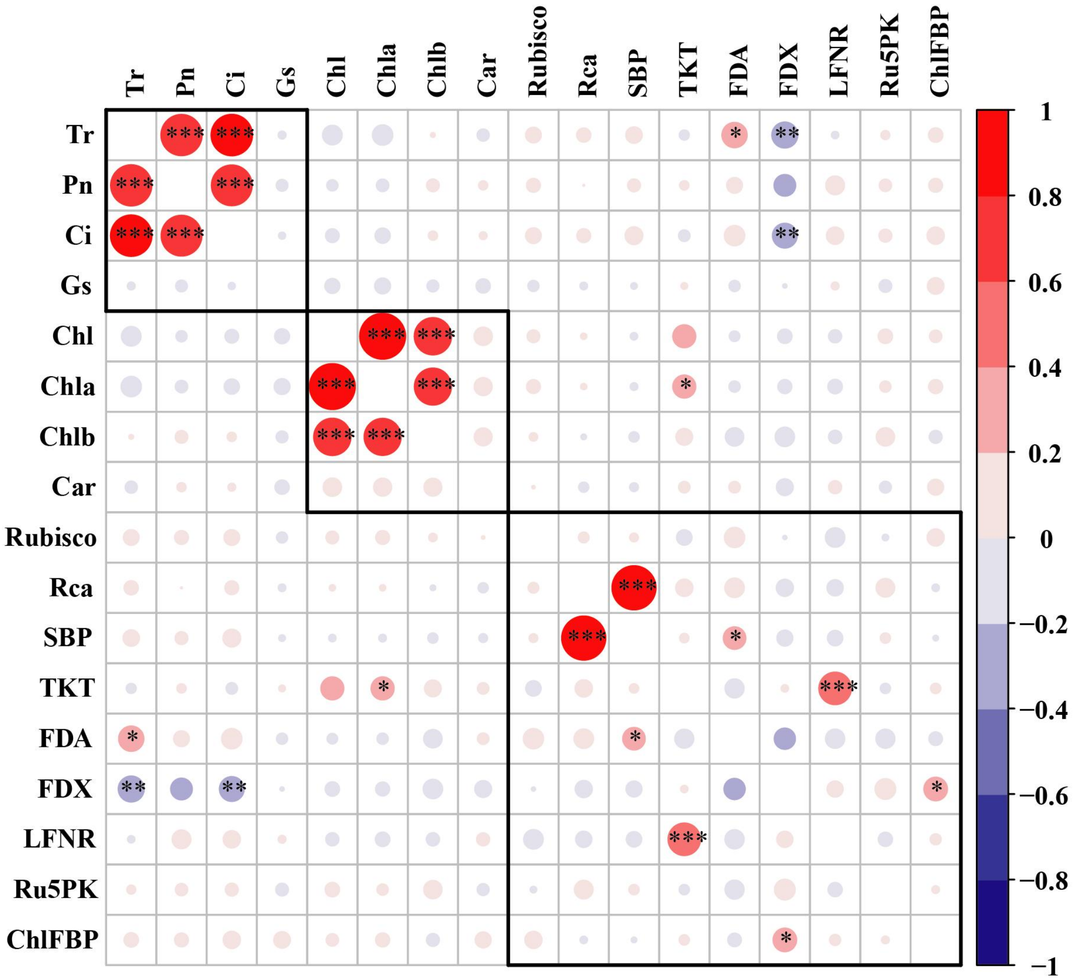

Supplementary Figure S1. Pearson correlation among 17 photosynthetic traits. The black box lines represent the same category of photosynthetic traits. ***$P <$ 0.001, **$P < 0.01$, *$P < 0.05$.

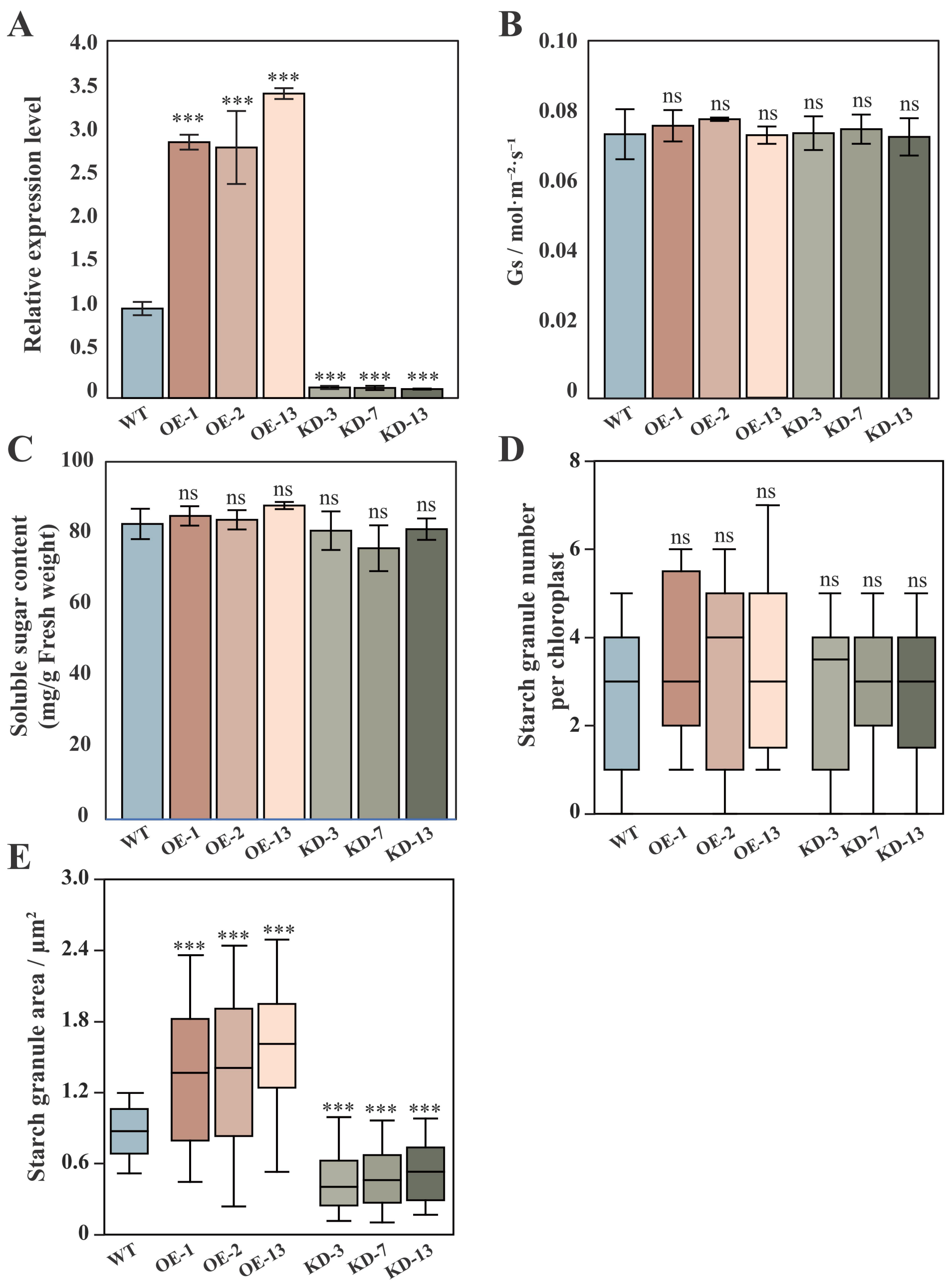

Supplementary Figure S2. The phenotypes of *PtoIRKI*-overexpressing (OE) and *ptoirki*-knockdown (KD) lines. A) Expression of *PtoIRKI* in transgenic poplar. The poplar *Actin* was used as an internal standard for data normalization. Values are means ± SD (n=3). **B)** Stomatal conductance (Gs) of the seventh leaves of WT, OE, and KD plants. Error bars are ± SD (n=3) **C)** Soluble sugar contents in the seventh leaves of WT, OE, and KD plants at 06:00. Data are means ± SD (n = 3). **D)** Number of starch granules per chloroplast. The average number of starch granules in 50 chloroplasts was measured in each plant. In box plots, center line represents the

median, box limits denote the upper and lower quartiles, whickers indicate the interquartile range, and dots are outliers. **E)** Area of each starch granule in WT, OE, and KD plants. The average area of 50 starch granules in each plant was calculated. In box plots, center line represents the median, box limits denote the upper and lower quartiles, whickers indicate the interquartile range, and dots are outliers. Differences were evaluated using two-tailed Student's *t*-tests. *$P < 0.05$; **$P < 0.01$; ***$P < 0.001$; ns, no significant difference.

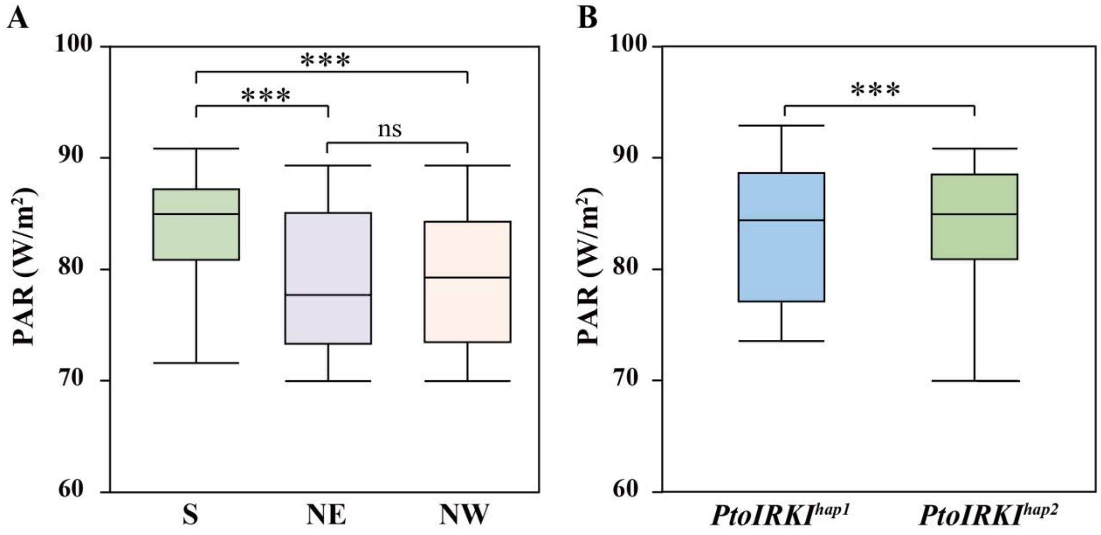

Supplementary Figure S3. Photosynthetically active radiation (PAR) across different geographical regions and among *PtoIRKI* haplotypes in *P. tomentosa.* A) PAR in three geographical distribution regions of *P. tomentosa.* NW, NE, and S represent northwest, northeast, and southern climate regions, respectively (n = 272). Differences were evaluated using one-way ANOVA and Tukey's test. ***$P < 0.001$; ns, no significant difference. In box plots, center line represents the median, box limits denote the upper and lower quartiles, whickers indicate the interquartile range, and dots are outliers. **B)** Differences in PAR among habitats associated with different *PtoIRKI* haplotypes (n = 272). In box plots, center line represents the median, box limits denote the upper and lower quartiles, whickers indicate the interquartile range, and dots are outliers. Differences were evaluated using two-tailed Student's *t*-tests. ***$P < 0.001$.

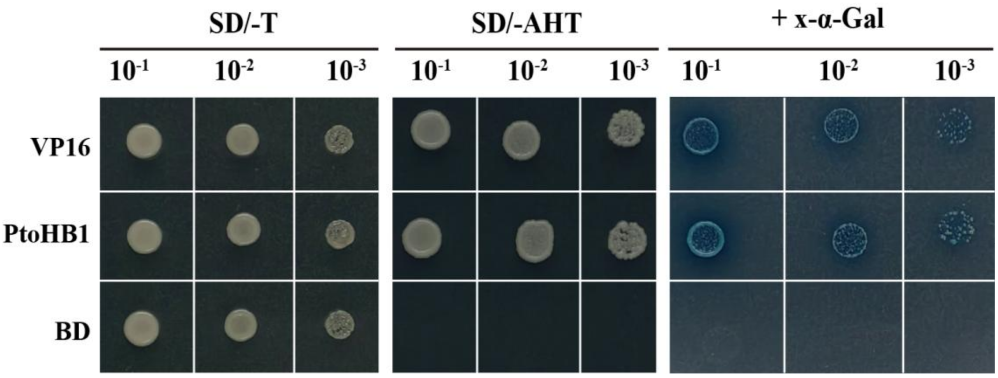

Supplementary Figure S4. Analysis of the transcriptional activation of *PtoHB1*. PtoHB1-pGBKT7 (PtoHB1) was used as the experimental group, and VP16 and pGBKT7 (BD) were used as the positive and negative controls respectively to verify the transcriptional activation activity of *PtoHB1*.

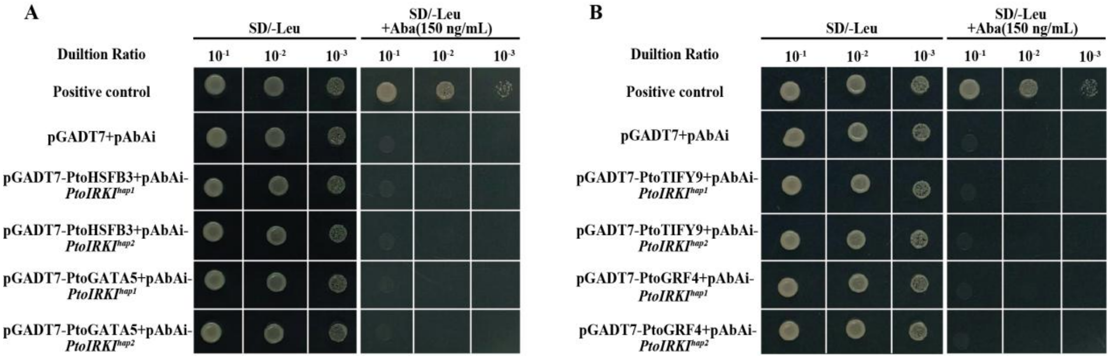

Supplementary Figure S5. Yeast one-hybrid (Y1H) assays of the top four (excluding PtoHB1) high-priority genes in the machine learning regression model with two haplotype promoters of *PtoIRKI*. Yeast one-hybrid assays examining the binding of PtoHSFB3 and PtoGATA5 **(A)**, PtoTIFY9 and PtoGRF4 **(B)** to the two *PtoIRKI* haplotype promoters. *PtoIRKI*^{hap1}: without the 12-bp insertion; *PtoIRKI*^{hap2}: with the 12-bp insertion.
